# The Effect of Immune Selection on the Structure of the Meningococcal Opa Protein Repertoire

**DOI:** 10.1371/journal.ppat.1000020

**Published:** 2008-03-14

**Authors:** Martin J. Callaghan, Caroline O. Buckee, Keith A. Jolley, Paula Kriz, Martin C. J. Maiden, Sunetra Gupta

**Affiliations:** 1 Department of Paediatrics, University of Oxford, Centre for Clinical Vaccinology and Tropical Medicine (CCVTM), Churchill Hospital, Oxford, United Kingdom; 2 Department of Zoology, University of Oxford, Oxford, United Kingdom; 3 Peter Medawar Building for Pathogen Research, Department of Zoology, University of Oxford, Oxford, United Kingdom; 4 National Reference Laboratory for Meningococcal Infections, National Institute of Public Health, Prague, Czech Republic; University of Toronto, Canada

## Abstract

The *opa* genes of the Gram negative bacterium *Neisseria meningitidis* encode Opacity-associated outer membrane proteins whose role is to promote adhesion to the human host tissue during colonisation and invasion. Each meningococcus contains 3–4 *opa* loci, each of which may be occupied by one of a large number of alleles. We analysed the Opa repertoire structure in a large, well-characterised collection of asymptomatically carried meningococci. Our data show an association between Opa repertoire and meningococcal lineages similar to that observed previously for meningococci isolated from cases of invasive disease. Furthermore, these Opa repertoires exhibit discrete, non-overlapping structure at a population level, and yet low within-repertoire diversity. These data are consistent with the predictions of a mathematical model of strong immune selection upon a system where identical alleles may occupy different loci.

## Author Summary

*Neisseria meningitidis* is a globally important pathogen that causes 2,000–3,000 cases of invasive meningococcal disease annually in the United Kingdom. The meningococcal Opa proteins are important in mediating adhesion to and invasion of human tissues, and are important for evasion of the host immune response. They are encoded by a repertoire of 3–4 genomic loci in each meningococcus and exhibit high levels of sequence diversity. Here we analyzed the Opa repertoires of a large, well-characterised, asymptomatically carried meningococcal isolate collection. We found that the Opa repertoires were specific to individual meningococcal genotypes, similar to that observed in isolates from cases of invasive disease. These repertoires exhibited discrete, non-overlapping structure at a population level, and yet low within-repertoire diversity. These data were consistent with the predictions of a mathematical model of strong immune selection, suggesting that the collective immune response of the host population shapes the antigenic diversity of the meningococcal Opa repertoire. This study provides new insights into Opa-mediated meningococcal pathogenesis and the effect of host population immunity on the biodiversity and population structure of bacterial pathogens. These data may also have implications for the design of new meningococcal vaccines based on surface proteins.

## Introduction

The Opacity (Opa) proteins of the bacterial pathogen *Neisseria meningitidis* mediate adhesion to and invasion of the human nasopharyngeal epithelium [1] *via* interaction with cell surface saccharides [2] and members of the carcinoembryonic antigen cell adhesion molecule (CEACAM) family of proteins [3],[4]. The *opa* gene repertoire comprises 3–4 loci per meningococcus (*opaA*, *opaB*, *opaD* and *opaJ*) [5]–[8]. These are constitutively transcribed and their expression is controlled by stochastic changes in a phase variable, pentameric repeat tract within the reading frame of the genes [9]. Varying numbers of *opa* loci may be expressed at different times and in different combinations, providing both functional flexibility and a possible mechanism for immune evasion.

Opa proteins are highly diverse [8],[10] with the majority of sequence changes localised in three regions which correspond to surface exposed loops in the proposed protein structure. It is thought that different sequences in the semivariable (SV) and two immunodominant hypervariable (HV) regions [10],[11] confer different receptor specificities to the protein [12],[13]. Diversity is generated by gene conversion, mosaicism and also modular exchange of variable regions, with the consequence that different *opa* loci in the same meningococcus may encode identical, similar or diverse HV regions [14],[15].

It has been shown that the Opa repertoire is highly structured among the hyperinvasive lineages of meningococci that are responsible for the majority of global disease [16]. Isolates from the same hyperinvasive clonal complexes (as defined by MLST) have been shown to possess similar and often identical Opa repertoires, despite being sampled from disparate geographical locations and temporal periods [8]. Little information is available, however, on the extent of the diversity of the Opa repertoire in carried populations of meningococci which contain the majority of meningococcal biodiversity. In this investigation, we analysed the Opa repertoires of a geographically and temporally related collection of asymptomatically carried meningococci to determine whether the association between clonal complexes and particular combinations of these adhesins, as observed in hyperinvasive lineages, was present in non-disease causing meningococci.

We analysed the data using a theoretical model of immune selection which incorporated the particular features of this antigenic system including its phase variable nature and the modular exchange of variable regions within genotypes. We found the patterns of diversity evident at both the population level and within individual repertoires to be indicative of strong immunological selection acting in addition to the forces of functional adaptation in influencing the structure of the Opa repertoire.

## Results

### Association between Opa repertoire and clonal complex

The four known *opa* loci were analysed in the 216 meningococcal isolates from a carried population sample from the Czech Republic: a total of 864 loci. In 784 loci (90.74%) an intact *opa* sequence was detected; these contained a total of 222 alleles (nucleotide *p* distance: 13.59%). These encoded 76 HV1 variants (amino acid *p* distance: 47.8%) which fell into 19 families and 93 HV2 variants (amino acid *p* distance: 37.6%) which fell into 21 families. A total of 212 *opa* loci were also analysed in a contemporaneous collection of 53 isolates from invasive disease. In 185 loci (87.26%) an *opa* sequence was detected, these contained a total of 75 alleles (nucleotide *p* distance: 14.26%). These encoded 41 HV1 variants (amino acid *p* distance: 48.4%) which fell into 15 families and 44 HV2 variants (amino acid *p* distance: 40.1%) which fell into 17 families.

In both the carriage and disease collections, we found that genetically related isolates, whether belonging to hyperinvasive clonal complexes or not, often had identical Opa repertoires (see Text S1 and Tables S1 and S2). For example, for the ST-11 complex, the *opa* gene alleleic repertoire *opaA* 83, *opaB* 11, *opaD* 132 and an insertionally inactivated *opaJ* locus was present in 27 of 32 carried isolates and 16 of 20 disease isolates. The remaining isolates from this complex in each collection had highly related repertoires, differing at only one or two loci. These repertoires were highly similar to those of isolates belonging to the same clonal complexes observed in a global collection of hyperinvasive meningococci (see Text S1 and Tables S1 and S2)[8].

### Population structure of Opa repertoires

**Figure 1:**
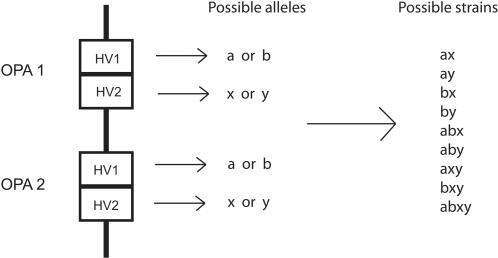
Schematic of the model setup. Each isolate has two opa loci, each having two HV regions (HV1 and HV2). Each HV region can express one of two possible variants, a or b for HV1 and x or y for HV2, which may be the same for both opa genes. There is no dose dependence; an isolate with locus 1 expressing ‘ax’ and locus 2 expressing ‘ax’ is considered to be just ‘ax’. This simple 2-locus system can be generalized to more loci without affecting the qualitative model outcome.

**Figure 2:**
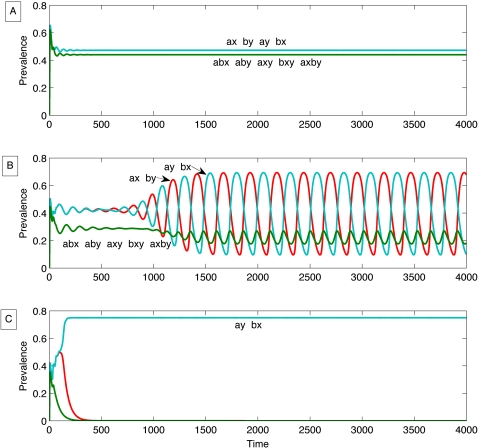
Different dynamics generated by the model. a) All strains coexist (no strain structure NSS), but strains expressing 3 and 4 epitopes are suppressed, γ = 0.65. b) Cyclical dynamics (cyclical strain structure CSS) with successive dominance of discordant sets of strain expressing 2 variants, and suppressed oscillations of strains expressing 3 and 4 alleles , γ = 0.83. c) The pathogen population is dominated by a subset of non-overlapping strains (DSS), each expressing two alleles, all other strains are suppressed, γ = 0.9.

The modular exchange of the immunodominant HV regions among different *opa* loci [14],[15] makes the system unusual in the context of immune selection, since the same hypervariable region variants may be present at multiple *opa* loci within the same isolate, as well as in different isolates. We extended a multi-locus mathematical model (see Materials and Methods for details) developed by Gupta *et al.* [17] to incorporate this feature by allowing the two HV regions (HV1 and HV2) at each locus to contain two possible amino acid sequence epitopes (‘a’ and ‘b’ for HV1 and ‘x’ and ‘y’ for HV2) as shown in Figure 1. Thus, possible combinations of HV regions in Opa proteins expressed by different meningococci could be: ‘ax’, ‘ay’, ‘bx’, ‘by’, ‘axy’, ‘bxy’, ‘abx’, ‘aby’, and ‘axby’. The behaviour of this model under different levels of immune selection is shown in Figure 2. These simulations indicate that the system shows a tendency to self organise at a population level into discrete antigenic types as the strength of immune selection increases, as previously observed [17] for multi-locus systems without modular exchange of variable regions. When immunological selection (as measured by cross-protection between pathogen types sharing variable regions) is weak, all antigenic types coexist at the similar abundances as shown in Figure 2a. By contrast, when immunological selection is high, a subset of two strains expressing two non-overlapping HV1/HV2 region combinations (for example, ‘ax’ and ‘by’) dominates, excluding all other strains, as exemplified by Figure 2c. Between these two extremes, we observe cyclical dynamics with strains expressing subsets of non-overlapping HV variants successively dominating the population (Figure 2b).

**Figure 3:**
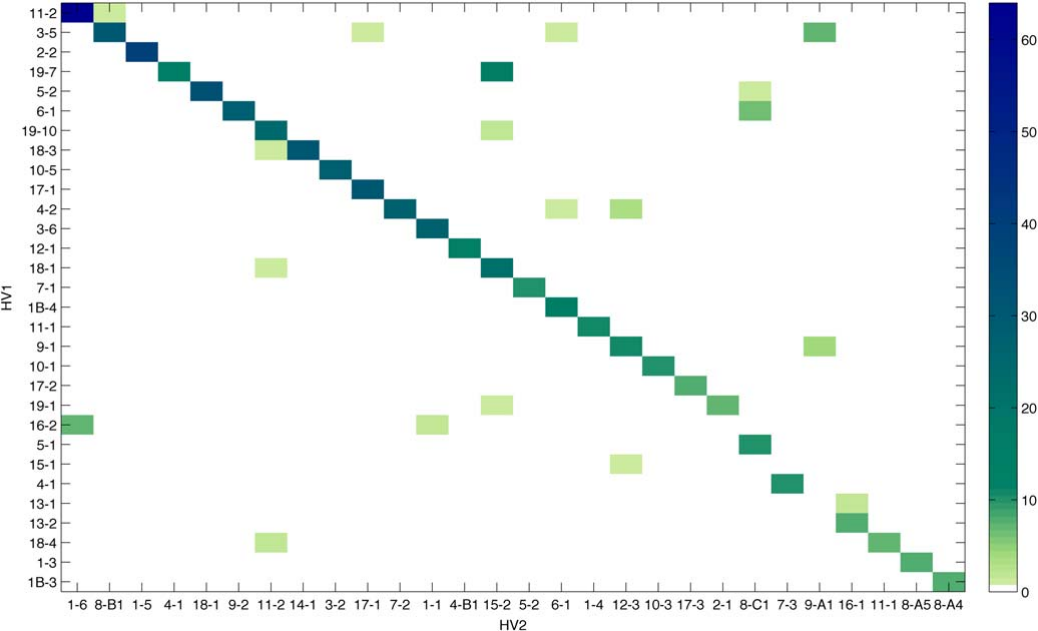
The combination of HV1-HV2 epitopes occurring in all carried isolates from the Czech Republic between March and June of 1993, presented as a heatmap. HV1 and HV2 epitopes are classified into alleles as described in the Materials and Methods section. The colours indicate the total number of observations of each particular combination. Each opa locus is treated independently, so each isolate can contribute more than one combination. (See Text S1 for an alternative representation of the data).

Figure 3 shows the combinations of HV1 and HV2 present at all loci for all isolates. Each opa locus is treated independently, so each isolate can contribute more than one combination. Variants for which only a single isolate was found were excluded from this analysis (see Table S3 for full details). To determine which of these population structures best described these data, a simple metric (f*) was developed to assess the extent of overlap between two epitopes among different isolates (see Materials and Methods for the derivation, and Text S1 for model validation): f* scores close to 1 indicate a highly non-overlapping structure, expected when cross-immunity is high, whereas scores close to 0 occur when strains have completely overlapping antigenic repertoires. Scores obtained from the *opa* loci in the dataset were compared to scores from housekeeping genes belonging to the same isolates. The f* metric for the data shown in Figure 3 is 0.9737, whereas pairwise comparisons of the housekeeping gene loci yielded a mean f* score of 0.3453 and a maximum of 0.4578. These scores indicate the non-overlapping nature of the Opa HV1/HV2 combinations as compared to the housekeeping loci, and reflect the diagonal pattern observed in the figure.

A total of 124 HV1/HV2 combinations were observed out of a possible total of 7068 (76 HV1 variants multiplied by 93 HV2 variants). Discrete, non-overlapping combinations of HV1 and HV2 are clearly dominant, despite the presence of rare combinations generated by frequent recombinational exchange. These observations support the model structure described above in which strong immune selection is responsible for structuring Opa repertoires.

### Diversity within Opa repertoires

Another important feature of the simulations presented in Figure 2 which is unique to a system with modular exchange between loci is that immune selection paradoxically leads to a reduction in diversity within individual opa repertoires. In other words, if more than one *opa* locus is expressed *in vivo*, selection will favour those strains expressing multiple loci encoding the same combination of HV regions, rather than different, more diverse variants. It is evident from Figure 2a that even under low levels of immune selection, the prevalence of strains expressing three or four antigenic determinants is suppressed. The magnitude of suppression increases with the degree of cross-protection (represented in the model by the parameter γ), such that these more diverse types are entirely absent in Figure 2c. This suppression occurs because strains expressing more than two HV variants are less likely to encounter hosts who have not previously been exposed to one or more of their epitopes, and are therefore at a disadvantage within the population. Thus, at very high levels of immune selection, we observe only meningococci that expressed a single *opa* locus, or multiple loci encoding the same combination of HV regions (i.e. ‘ax’ at locus 1 and ‘ax’ at locus 2). This is because the pathogen population, and therefore the background of host immunity, is dominated by two non-overlapping strains, say ‘ax’ and ‘by’, so that more diverse strains (such as ‘axy’) are more likely to be recognized by hosts who have encountered either one of the dominant strains.

**Figure 4:**
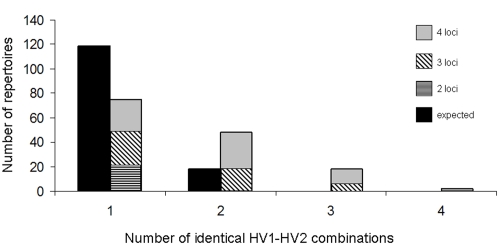
Observed and expected numbers of Opa loci per repertoire with identical HV1-HV2 combinations. Only unique repertoires were included, in order to control for prevalent repertoires, and the x-axis shows how many of these loci have identical HV combinations. The ‘random’ distribution was generated using the same number of HV combinations found in the data, and these were randomly distributed among isolates. Note that the deviation from the data would be even greater if the HV combinations were truly randomized, ie. if all combinations had the same probability of occurring in a given repertoire. The observed number of isolates with two or more of the same HV combination departs significantly from the expected number (p<0.0001).

To investigate the effect of host immunity on the structuring of the HV region repertoire diversity in individual isolates, we analysed the HV combinations of different Opa proteins within the same isolate for those that had full *opa* sequences at more than one locus (not including those that had frame-shift mutations or insertional inactivations). Figure 4 shows the proportion of isolates with identical HV1/HV2 combinations at different *opa* loci within the same isolate, compared to a hypothetical pathogen population in which the same combinations found in the data were distributed randomly within and among isolates. Only unique Opa repertoires were included in the analysis, to control for bias due to particularly prevalent sequence types and clonal complexes.

Our results showed that significantly more isolates contained two or more of the same HV1/HV2 combination than would be expected by chance given the same overall prevalence of variants (p<0.0001). Furthermore, they were not always the same HV1/HV2 combinations that were identical, with 28 different combinations occurring more than once within isolates. Finally, the probability that two or more were identical increased with the number of *opa* loci at which a full length *opa* allele was detected for each isolate (see Figure 4).

## Discussion

The Opa repertoire structure observed in the carried meningococci from the Czech Republic, and its relationship to clonal complex, was similar to that previously described in an isolate collection representing the diversity of meningococci causing disease globally in the latter half of the 20^th^ century [8]. This does not imply that the Opa repertoire has no role in meningococcal pathogenesis since other factors, such as differences in expression patterns among meningococci and host susceptibility, are likely to influence the outcome of infection.

Despite evidence for extensive recombination of *opa* loci among meningococci, only a fraction of all possible combinations of HV1 and HV2 were observed. . These combinations exhibited a non-random and non-overlapping structure, which was consistent with a model of immunological selection in which competition between pathogen types leads to a pathogen population dominated by non-overlapping combinations of antigenic variants [17]–[19]. The low frequency off-diagonal elements shown in Figure 3 may be attributed either to the point prevalent nature of the data set (ie. that these combination are shortlived) or reflect the fact that certain variants possess immunological similarities, and are therefore equally likely to occur in combination with certain others. It is also possible that there are functional constraints in operation here since particular HV1/HV2 combinations influence receptor tropism and potentially also avidity [12],[13],[20]. It has been suggested that expression of CEACAM on host cell surfaces may allow evasion of antibody responses by Opa-mediated entry into epithelial cells [4] and modulation of the host immune responses by interaction with CD4+ T cells [21]. The specificity of these interactions is likely to constrain allowable HV1/HV2 combinations and may explain why particular combinations are entirely absent in our data.

Non-overlapping patterns of epitope combinations have also been observed among meningococcal PorA variable regions [17],[19]. Unlike PorA however, the Opa repertoire is a four-locus system [5],[8], and has been suggested to play a role in immune evasion [22],[23]. For the Opa proteins, individual repertoires exhibited more identical HV variants than would be expected under the assumption that antigenic diversity of a surface component prolongs infection (Figure 4). This result is, however, consistent with the predictions of a mathematical model of strong immune selection upon a system where identical alleles may occupy different loci. Within this framework, isolates expressing diverse repertoires are at a disadvantage because they are more likely to encounter hosts with previous exposure to one or more of their epitopes. This results in selection for identical variants at multiple loci: in other words, a reduction in the diversity of the Opa repertoires within individual meningococci. The prevalence of identical Opa variants within repertoires implies that multiple *opa* loci are expressed *in vivo*; if expression were restricted to a single *opa* locus, there would be no selective disadvantage of carrying a diverse repertoire. An alternative explanation for the low within-repertoire diversity is that identical HV combinations reflect genetic duplication events that are followed by specialisation of duplicates for new functions [24],[25].

An exception to the pattern of diversity within the Opa repertoires in most clonal complexes was that of the ST-11 complex which did not have any identical HV variants among its loci. This may be due to the recent entry and rapid spread of this clonal complex into the population of the Czech Republic, where it was responsible for a rapid increase in the incidence, mortality and morbidity of invasive meningococcal disease in 1993 [26]. Retrospective monitoring of isolates since 1970 suggested that this strain was not present in the country before 1993 and consequently the Czech population may have been immunologically naïve, allowing these meningococci to spread through the population. Thus, high Opa repertoire diversity may be selectively advantageous for the invasion of new communities of hosts with variable immunological backgrounds. During prolonged carriage in the same host population however, increased diversity may become costly as the proportion of immunologically naïve hosts decreases. This would inevitably cause a reduction in the range of receptor tropism, but this would be offset by the gain in probability of transmission. To date, the majority of Opa proteins tested bind at least CEACAM1 [27], suggesting that the repertoire retains binding of this major receptor.

Intriguingly, the number of *opa* loci differ among the *Neisseria* species, with 3–4 in Neisseria meningitidis [6],[7], 11–12 loci in *Neisseria gonorrhoeae* [28] and two in *Neisseria lactamica* [29]. The reasons for these differences are unclear, but our analyses in this study suggest a theory based on population prevalence and immunological cross-protection. For example, whereas *N. meningitidis* is transmitted by aerosol inhalation, *N. gonorrhoeae* is transmitted sexually and consequently has a much lower population prevalence. The likelihood of *N. gonorrhoeae* encountering an immunologically naïve host may be much higher, therefore, and the diversity-reducing effect from the host population's immunological responses less pronounced than for the meningococcus. A more diverse Opa repertoire with more loci may be more advantageous in these circumstances. Further information on the antigenic diversity of the gonococcal Opa repertoire and immunological responses against both pathogenic *Neisseria* species would be required to test this hypothesis.

In conclusion, this analysis demonstrates that particular Opa repertoires are associated with meningococcal clonal complexes irrespective of geographic or temporal sampling, whether isolated from asymptomatic carriers or invasive disease cases. The repertoires exhibit discrete, non-overlapping structure on a population level and low within-repertoire diversity, indicating that immune selection plays an important role in shaping Opa repertoires.

## Materials and Methods

### Isolate collection, determination of *opa* gene sequences and variable region assignment

A total of 216 meningococcal isolates were obtained from an asymptomatically carried population of meningococci collected in the Czech Republic between March and June of 1993 [30]. A full description of these meningococci, including year and location of isolation, MLST and antigen gene sequencing data appears online at http://pubmlst.org. Genomic DNA was prepared by culturing isolates as previously described [30] before extracting with a DNA mini kit (Qiagen, Crawley, UK) according to the manufacturer's instructions. The *opa* loci were isolated in separate, locus-specific PCR amplifications, their nucleotide sequences determined at least once on each strand and their variable regions identified as previously described [8]. Nucleotide and amino acid sequence data are available in an online database located at http://neisseria.org/nm/typing/opa/. For analyses of diversity, by uncorrected nucleotide or amino acid percentage (*p*) distance, sequences were aligned and diversity calculated using the program DAMBE: Data Analysis and Molecular Biology and Evolution [31].

### Derivation of the f* metric

A non-overlapping strain structure results in a matrix of allelic associations between two antigenic loci in which each allele at locus 1 should be predominantly associated with only one allele at locus 2, and vice versa. This means that the most prevalent strains should dominate both the ‘row’ and ‘column’ of their allelic association matrix. The level of overlap within such a matrix can therefore be measured by assessing the dominance of the most prevalent allele combinations;
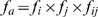
The dominance of the most prevalent allele combination in each column (*f_a_*) is calculated, where locus 1 expresses allele *i,* and locus 2 expresses allele *j*. *f_i_* is the frequency of the most prevalent strain expressing allele *i* at locus 1, with respect to all strains expressing that allele (ie. the ‘column’ dominance), *f_j_* is the frequency of that strain with respect to all strains expressing allele *j* at locus 2 (ie. the ‘row’ dominance), and *f_ij_* is the frequency of allele combination *ij* overall. These are calculated as follows:
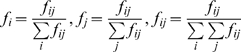
The sum over all *f_a_* gives the overall overlap between two loci:
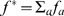
such that f* varies between 0 and 1. For a completely non-overlapping matrix, with no combinations found except for those that do not overlap, f* will be exactly one. As this structuring breaks down, the f* score will decrease rapidly.

### Model setup

Three differential equations, based on a model by Gupta *et al.* [17],[18], describe the system:
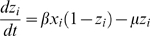


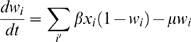


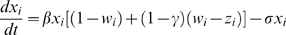
The model states that once infected with a particular strain, the host gains partial immunity to any other strains expressing shared antigenic determinants (the subset of strains *i'* above) as specified by the parameter γ. For each strain *i*, the host population consists of three overlapping compartments; the proportion infectious to other hosts, *x_i_*; the proportion exposed (and therefore immune) to strain *i*, *z_i_*, and the proportion exposed to any strain sharing antigenic determinants with *i*, *w_i_*. It was assumed that the duration of infectiousness (1/σ) was short compared to the average host life-span (1/μ), and that immunity was life-long. All strains were assumed to have the same transmission coefficient, β. The effect of recombination was not explicitly included in the model, however all possible strains were present from the start in order to investigate the competitive interactions between them. Note that in this model there was no dose-dependence; two loci expressing Opa proteins with identical HV regions was taken as being the same as if only one locus expressed the protein.

## Supporting Information

Table S1 *opa* repertoires of meningococci isolated from asymptomatic carriage in the Czech Republic during 1993. ST: multilocus sequence typing (MLST) sequence type, CC: MLST clonal complex SV: semi variable region variant, HV1: first hypervariable region variant, HV2: second hypervariable region variant, ND: opa sequence not detected, ININ: insertional inactivation of opa locus by insertion sequence-like element, FSM: opa locus present but non-functional due to frameshift mutation.
(.097 MB DOC)

Table S2 *opa* repertoires of meningococci isolated from invasive disease in the Czech Republic during 1993.
ST: multilocus sequence typing (MLST) sequence type, CC: MLST clonal complex SV: semi variable region variant, HV1: first hypervariable region variant, HV2: second hypervariable region variant, ND: opa sequence not detected, ININ: insertional inactivation of *opa* locus by insertion sequence-like element, FSM: opa locus present but non-functional due to frameshift mutation.
(0.19 MB DOC)

Table S3 The combination of all HV1-HV2 epitopes occurring in all carried isolates from the Czech Republic between March and June of 1993.
(Figure 3 in the text does not contain variants for which there is only a single isolate found). HV1 and HV2 epitopes are classified into alleles as described in the methods section.
(0.03 MB DOC)

Text S1 Supplementary material
(0.10 MB DOC)
