# Supplementary material for: The Effect of Immune Selection on the Structure of the Meningococcal Opa Protein Repertoire
Source: PLoS Pathog. 2008 Mar 14;4(3):e1000020. doi: 10.1371/journal.ppat.1000020 (PMC2265424; doi:10.1371/journal.ppat.1000020)
Supplement: Table S1 — opa repertoires of meningococci isolated from asymptomatic carriage in the Czech Republic during 1993. ST: multilocus sequence typing (MLST) sequence type, CC: MLST clonal complex SV: semi variable region variant, HV1: first hypervariable region variant, HV2: second hypervariable region variant, ND: opa sequence not detected, ININ: insertional inactivation of opa locus by insertion sequence-like element, FSM: opa locus present but non-functional due to frameshift mutation. (.097 MB DOC) [file ppat.1000020.s001.doc]

**Supplementary Table 1.** *opa* repertoires of meningococci isolated from asymptomatic carriage in the Czech Republic during 1993. ST: multilocus sequence typing (MLST) sequence type, CC: MLST clonal complex SV: semi variable region variant, HV1: first hypervariable region variant, HV2: second hypervariable region variant, ND: opa sequence not detected, ININ: insertional inactivation of *opa* locus by insertion sequence-like element, FSM: opa locus present but non-functional due to frameshift mutation.

| **ST** | **CC** | **Isolate** | **opaA** | **SV** | **HV1** | **HV2** | **opaB** | **SV** | **HV1** | **HV2** | **opaD** | **SV** | **HV1** | **HV2** | **opaJ** | **SV** | **HV1** | **HV2** |
| --- | --- | --- | --- | --- | --- | --- | --- | --- | --- | --- | --- | --- | --- | --- | --- | --- | --- | --- |
| 11 | 11 | 0022/93 | 83 | 3-4 | 5-2 | 18-1 | 11 | 3-1 | 18-3 | 14-1 | 132 | 2-2 | 11-2 | 1-6 | ININ | ININ | ININ | ININ |
| 11 | 11 | 0023/93 | 83 | 3-4 | 5-2 | 18-1 | 11 | 3-1 | 18-3 | 14-1 | 132 | 2-2 | 11-2 | 1-6 | ININ | ININ | ININ | ININ |
| 11 | 11 | 0025/93 | 83 | 3-4 | 5-2 | 18-1 | 11 | 3-1 | 18-3 | 14-1 | 132 | 2-2 | 11-2 | 1-6 | ININ | ININ | ININ | ININ |
| 11 | 11 | 0035/93 | 83 | 3-4 | 5-2 | 18-1 | 11 | 3-1 | 18-3 | 14-1 | 132 | 2-2 | 11-2 | 1-6 | ININ | ININ | ININ | ININ |
| 11 | 11 | 0036/93 | 83 | 3-4 | 5-2 | 18-1 | 11 | 3-1 | 18-3 | 14-1 | 132 | 2-2 | 11-2 | 1-6 | ININ | ININ | ININ | ININ |
| 11 | 11 | 0037/93 | 83 | 3-4 | 5-2 | 18-1 | 11 | 3-1 | 18-3 | 14-1 | 132 | 2-2 | 11-2 | 1-6 | ININ | ININ | ININ | ININ |
| 11 | 11 | 0039/93 | 83 | 3-4 | 5-2 | 18-1 | 11 | 3-1 | 18-3 | 14-1 | 132 | 2-2 | 11-2 | 1-6 | ININ | ININ | ININ | ININ |
| 11 | 11 | 0042/93 | 83 | 3-4 | 5-2 | 18-1 | 11 | 3-1 | 18-3 | 14-1 | 132 | 2-2 | 11-2 | 1-6 | ININ | ININ | ININ | ININ |
| 11 | 11 | 0043/93 | 83 | 3-4 | 5-2 | 18-1 | 11 | 3-1 | 18-3 | 14-1 | 132 | 2-2 | 11-2 | 1-6 | ININ | ININ | ININ | ININ |
| 11 | 11 | 0045/93 | 83 | 3-4 | 5-2 | 18-1 | 18 | 3-1 | 18-3 | 14-1 | 132 | 2-2 | 11-2 | 1-6 | ININ | ININ | ININ | ININ |
| 11 | 11 | 0046/93 | 83 | 3-4 | 5-2 | 18-1 | 11 | 3-1 | 18-3 | 14-1 | 132 | 2-2 | 11-2 | 1-6 | ININ | ININ | ININ | ININ |
| 11 | 11 | 0073/93 | 83 | 3-4 | 5-2 | 18-1 | 11 | 3-1 | 18-3 | 14-1 | 132 | 2-2 | 11-2 | 1-6 | ININ | ININ | ININ | ININ |
| 11 | 11 | 0074/93 | 83 | 3-4 | 5-2 | 18-1 | 11 | 3-1 | 18-3 | 14-1 | 132 | 2-2 | 11-2 | 1-6 | ININ | ININ | ININ | ININ |
| 11 | 11 | 0079/93 | 83 | 3-4 | 5-2 | 18-1 | 11 | 3-1 | 18-3 | 14-1 | 132 | 2-2 | 11-2 | 1-6 | ININ | ININ | ININ | ININ |
| 11 | 11 | 0093/93 | 83 | 3-4 | 5-2 | 18-1 | 11 | 3-1 | 18-3 | 14-1 | 132 | 2-2 | 11-2 | 1-6 | ININ | ININ | ININ | ININ |
| 11 | 11 | 0095/93 | 83 | 3-4 | 5-2 | 18-1 | 11 | 3-1 | 18-3 | 14-1 | 132 | 2-2 | 11-2 | 1-6 | ININ | ININ | ININ | ININ |
| 11 | 11 | 0096/93 | 83 | 3-4 | 5-2 | 18-1 | 11 | 3-1 | 18-3 | 14-1 | 132 | 2-2 | 11-2 | 1-6 | ININ | ININ | ININ | ININ |
| 11 | 11 | 0099/93 | 83 | 3-4 | 5-2 | 18-1 | 11 | 3-1 | 18-3 | 14-1 | 132 | 2-2 | 11-2 | 1-6 | ININ | ININ | ININ | ININ |
| 11 | 11 | 0101/93 | 83 | 3-4 | 5-2 | 18-1 | 11 | 3-1 | 18-3 | 14-1 | 132 | 2-2 | 11-2 | 1-6 | ININ | ININ | ININ | ININ |
| 11 | 11 | 0102/93 | 83 | 3-4 | 5-2 | 18-1 | 11 | 3-1 | 18-3 | 14-1 | 132 | 2-2 | 11-2 | 1-6 | ININ | ININ | ININ | ININ |
| 11 | 11 | 0103/93 | 83 | 3-4 | 5-2 | 18-1 | 11 | 3-1 | 18-3 | 14-1 | 132 | 2-2 | 11-2 | 1-6 | ININ | ININ | ININ | ININ |
| 11 | 11 | 0109/93 | 83 | 3-4 | 5-2 | 18-1 | 11 | 3-1 | 18-3 | 14-1 | 132 | 2-2 | 11-2 | 1-6 | ININ | ININ | ININ | ININ |
| 11 | 11 | 0113/93 | 83 | 3-4 | 5-2 | 18-1 | 11 | 3-1 | 18-3 | 14-1 | 132 | 2-2 | 11-2 | 1-6 | ININ | ININ | ININ | ININ |
| 11 | 11 | 0115/93 | 83 | 3-4 | 5-2 | 18-1 | 11 | 3-1 | 18-3 | 14-1 | 132 | 2-2 | 11-2 | 1-6 | ININ | ININ | ININ | ININ |
| 11 | 11 | 0119/93 | 83 | 3-4 | 5-2 | 18-1 | 11 | 3-1 | 18-3 | 14-1 | 132 | 2-2 | 11-2 | 1-6 | ININ | ININ | ININ | ININ |
| 11 | 11 | 0270/93 | 83 | 3-4 | 5-2 | 18-1 | 11 | 3-1 | 18-3 | 14-1 | 132 | 2-2 | 11-2 | 1-6 | ININ | ININ | ININ | ININ |
| 11 | 11 | 0272/93 | 354 | 3-4 | 3-10 | 8-B1 | 11 | 3-1 | 18-3 | 14-1 | 132 | 2-2 | 11-2 | 1-6 | ININ | ININ | ININ | ININ |
| 11 | 11 | 0381/93 | 83 | 3-4 | 5-2 | 18-1 | ININ | ININ | ININ | ININ | 132 | 2-2 | 11-2 | 1-6 | ININ | ININ | ININ | ININ |
| 11 | 11 | 0383/93 | 83 | 3-4 | 5-2 | 18-1 | ININ | ININ | ININ | ININ | 132 | 2-2 | 11-2 | 1-6 | ININ | ININ | ININ | ININ |
| 11 | 11 | 0395/93 | 83 | 3-4 | 5-2 | 18-1 | 11 | 3-1 | 18-3 | 14-1 | 132 | 2-2 | 11-2 | 1-6 | ININ | ININ | ININ | ININ |
| 18 | 18 | 0016/93 | 89 | 2-2 | 19-7 | 4-B2 | 8 | 2-2 | 11-1 | 5-1 | 43 | 2-2 | 15-1 | 13-1 | 87 | 2-2 | 11-1 | 1-4 |
| 18 | 18 | 0089/93 | 89 | 2-2 | 19-7 | 4-B2 | 8 | 2-2 | 11-1 | 5-1 | 43 | 2-2 | 15-1 | 13-1 | 87 | 2-2 | 11-1 | 1-4 |
| 36 | - | 0246/93 | 109 | 2-2 | 19-2 | 14-2 | 54 | 2-1 | 5-2 | 18-1 | 135 | 4-3 | 16-1 | 1-2 | ND | ND | ND | ND |
| 44 | 44 | 0069/93 | 97 | 4-3 | 19-10 | 11-4 | 14 | 2-2 | 18-1 | 15-2 | 14 | 2-2 | 18-1 | 15-2 | 213 | 2-1 | 3-6 | 1-1 |
| 44 | 44 | 0213/93 | 24 | 4-3 | 19-10 | 11-2 | 14 | 2-2 | 18-1 | 15-2 | 14 | 2-2 | 18-1 | 15-2 | 213 | 2-1 | 3-6 | 1-1 |
| 44 | 44 | 0214/93 | 24 | 4-3 | 19-10 | 11-2 | 43 | 2-2 | 15-1 | 13-1 | ND | ND | ND | ND | 213 | 2-1 | 3-6 | 1-1 |
| 44 | 44 | 0244/93 | 53 | 3-2 | 15-1 | 13-2 | 14 | 2-2 | 18-1 | 15-2 | 14 | 2-2 | 18-1 | 15-2 | 353 | 5-2 | 16-2 | 1-1 |
| 44 | 44 | 0253/93 | 113 | 2-2 | 10-1 | 10-3 | ND | ND | ND | ND | 113 | 2-2 | 10-1 | 10-3 | 213 | 2-1 | 3-6 | 1-1 |
| 44 | 44 | 0255/93 | 113 | 2-2 | 10-1 | 10-3 | 181 | 2-2 | 10-1 | 10-3 | 113 | 2-2 | 10-1 | 10-3 | 213 | 2-1 | 3-6 | 1-1 |
| 44 | 44 | 0307/93 | 24 | 4-3 | 19-10 | 11-2 | 14 | 2-2 | 18-1 | 15-2 | 14 | 2-2 | 18-1 | 15-2 | 213 | 2-1 | 3-6 | 1-1 |
| 44 | 44 | 0320/93 | 122 | 4-3 | 19-10 | 11-3 | 14 | 2-2 | 18-1 | 15-2 | 113 | 2-2 | 10-1 | 10-3 | 213 | 2-1 | 3-6 | 1-1 |
| 53 | 53 | 0066/93 | 30 | 2-1 | 17-2 | 17-3 | 25 | 2-3 | 19-1 | 2-1 | 25 | 2-3 | 19-1 | 2-1 | 132 | 2-2 | 11-2 | 1-6 |
| 53 | 53 | 0083/93 | FSM | FSM | FSM | FSM | 30 | 2-1 | 17-2 | 17-3 | ND | ND | ND | ND | 230 | 2-2 | 11-2 | 1-6 |
| 53 | 53 | 0252/93 | 183 | 2-1 | 17-2 | 17-6 | 56 | 4-2 | 14-1 | 4-A1 | ND | ND | ND | ND | 132 | 2-2 | 11-2 | 1-6 |
| 53 | 53 | 0269/93 | 30 | 2-1 | 17-2 | 17-3 | 30 | 2-1 | 17-2 | 17-3 | 30 | 2-1 | 17-2 | 17-3 | 230 | 2-2 | 11-2 | 1-6 |
| 53 | 53 | 0410/93 | 30 | 2-1 | 17-2 | 17-3 | 30 | 2-1 | 17-2 | 17-3 | 30 | 2-1 | 17-2 | 17-3 | ND | ND | ND | ND |
| 53 | 53 | 0422/93 | 204 | 2-2 | 5-4 | 8-C1 | 194 | 2-1 | 17-2 | 17-6 | ND | ND | ND | ND | 157 | 2-7 | 2-2 | 1-5 |
| 53 | 53 | 0426/93 | 183 | 2-1 | 17-2 | 17-6 | 25 | 2-3 | 19-1 | 2-1 | ND | ND | ND | ND | 132 | 2-2 | 11-2 | 1-6 |
| 81 | - | 0054/93 | 13 | 2-1 | 17-1 | 17-1 | 21 | 2-1 | 18-2 | 15-3 | 143 | 2-2 | 11-2 | 1-6 | 215 | 3-2 | 18-2 | 15-1 |
| 82 | 254 | 0302/93 | 166 | 2-2 | 19-3 | 2-1 | 65 | 2-2 | 19-3 | 2-1 | 166 | 2-2 | 19-3 | 2-1 | 231 | 2-2 | 4-1 | 7-3 |
| 83 | 231 | 0230/93 | 94 | 2-2 | 3-5 | 8-B1 | 47 | 4-3 | 17-1 | 17-1 | 155 | 4-2 | 1B-2 | 5-7 | 135 | 4-3 | 16-1 | 1-2 |
| 84 | 92 | 0065/93 | 94 | 2-2 | 3-5 | 8-B1 | 13 | 2-1 | 17-1 | 17-1 | 139 | 2-6 | 11-2 | 1-6 | 219 | 2-2 | 3-5 | 8-B2 |
| 85 | - | 0199/93 | 24 | 4-3 | 19-10 | 11-2 | ND | ND | ND | ND | 99 | 3-2 | 4-1 | 7-3 | 211 | 5-2 | 16-2 | 1-6 |
| 85 | - | 0200/93 | 99 | 3-2 | 4-1 | 7-3 | 36 | 2-1 | 7-1 | 5-2 | 99 | 3-2 | 4-1 | 7-3 | 211 | 5-2 | 16-2 | 1-6 |
| 85 | - | 0202/93 | 99 | 3-2 | 4-1 | 7-3 | 36 | 2-1 | 7-1 | 5-2 | 99 | 3-2 | 4-1 | 7-3 | 211 | 5-2 | 16-2 | 1-6 |
| 85 | - | 0242/93 | 99 | 3-2 | 4-1 | 7-3 | 36 | 2-1 | 7-1 | 5-2 | 99 | 3-2 | 4-1 | 7-3 | 211 | 5-2 | 16-2 | 1-6 |
| 85 | - | 0298/93 | 117 | 3-2 | 7-1 | 5-2 | 36 | 2-1 | 7-1 | 5-2 | 99 | 3-2 | 4-1 | 7-3 | 211 | 5-2 | 16-2 | 1-6 |
| 86 | - | 0251/93 | 111 | 4-2 | 14-1 | 4-A2 | 25 | 2-3 | 19-1 | 2-1 | 135 | 4-3 | 16-1 | 1-2 | 111 | 4-2 | 14-1 | 4-A2 |
| 87 | - | 0209/93 | 86 | 4-2 | 12-1 | 4-B1 | 40 | 5-1 | 1A-3 | 8-A1 | 145 | 4-2 | 12-2 | 21-1 | 40 | 5-1 | 1A-3 | 8-A1 |
| 88 | - | 0205/93 | 100 | 2-2 | 19-4 | 4-1 | 39 | 2-2 | 5-1 | 8-C1 | 132 | 2-2 | 11-2 | 1-6 | 223 | 4-3 | 19-4 | 4-A3 |
| 89 | - | 0238/93 | 107 | 2-2 | 19-7 | 4-1 | 51 | 2-2 | 1B-1 | 18-1 | 159 | 2-2 | 1B-1 | 10-4 | 227 | 2-2 | 19-7 | 4-A3 |
| 89 | - | 0309/93 | 68 | 4-3 | 19-4 | 4-1 | 68 | 4-3 | 19-4 | 4-1 | 68 | 4-3 | 19-4 | 4-1 | 232 | 4-3 | 19-4 | 4-2 |
| 90 | - | 0265/93 | 207 | 4-2 | 19-10 | 11-3 | 59 | 2-2 | 19-10 | 11-3 | 161 | 2-2 | 11-1 | 1-4 | 228 | 2-2 | 19-10 | 11-3 |
| 91 | 92 | 0334/93 | 94 | 2-2 | 3-5 | 8-B1 | 13 | 2-1 | 17-1 | 17-1 | 139 | 2-6 | 11-2 | 1-6 | 94 | 2-2 | 3-5 | 8-B1 |
| 92 | 92 | 0030/93 | 94 | 2-2 | 3-5 | 8-B1 | 13 | 2-1 | 17-1 | 17-1 | 139 | 2-6 | 11-2 | 1-6 | 94 | 2-2 | 3-5 | 8-B1 |
| 92 | 92 | 0059/93 | 94 | 2-2 | 3-5 | 8-B1 | 24 | 4-3 | 19-10 | 11-2 | 146 | 2-6 | 11-2 | 1-6 | 94 | 2-2 | 3-5 | 8-B1 |
| 92 | 92 | 0085/93 | 94 | 2-2 | 3-5 | 8-B1 | 13 | 2-1 | 17-1 | 17-1 | 139 | 2-6 | 11-2 | 1-6 | 94 | 2-2 | 3-5 | 8-B1 |
| 92 | 92 | 0198/93 | 94 | 2-2 | 3-5 | 8-B1 | 35 | 2-2 | 17-1 | 17-1 | 139 | 2-6 | 11-2 | 1-6 | 221 | 2-2 | 3-5 | 8-B1 |
| 92 | 92 | 0218/93 | 102 | 2-1 | 19-7 | 14-2 | 13 | 2-1 | 17-1 | 17-1 | 139 | 2-6 | 11-2 | 1-6 | 224 | 2-2 | 3-4 | 8-B3 |
| 92 | 92 | 0220/93 | 102 | 2-1 | 19-7 | 14-2 | 13 | 2-1 | 17-1 | 17-1 | 139 | 2-6 | 11-2 | 1-6 | 224 | 2-2 | 3-4 | 8-B3 |
| 92 | 92 | 0243/93 | 13 | 2-1 | 17-1 | 17-1 | 13 | 2-1 | 17-1 | 17-1 | 139 | 2-6 | 11-2 | 1-6 | 94 | 2-2 | 3-5 | 8-B1 |
| 92 | 92 | 0276/93 | 94 | 2-2 | 3-5 | 8-B1 | 13 | 2-1 | 17-1 | 17-1 | 139 | 2-6 | 11-2 | 1-6 | 94 | 2-2 | 3-5 | 8-B1 |
| 92 | 92 | 0295/93 | 94 | 2-2 | 3-5 | 8-B1 | 13 | 2-1 | 17-1 | 17-1 | 163 | 2-2 | 11-2 | 1-6 | 94 | 2-2 | 3-5 | 8-B1 |
| 92 | 92 | 0323/93 | 94 | 2-2 | 3-5 | 8-B1 | 13 | 2-1 | 17-1 | 17-1 | 94 | 2-2 | 3-5 | 8-B1 | 94 | 2-2 | 3-5 | 8-B1 |
| 92 | 92 | 0338/93 | 94 | 2-2 | 3-5 | 8-B1 | 13 | 2-1 | 17-1 | 17-1 | 139 | 2-6 | 11-2 | 1-6 | 94 | 2-2 | 3-5 | 8-B1 |
| 92 | 92 | 0411/93 | 159 | 2-2 | 1B-1 | 10-4 | 13 | 2-1 | 17-1 | 17-1 | 139 | 2-6 | 11-2 | 1-6 | 240 | 2-2 | 1B-1 | 8-B1 |
| 92 | 92 | 0424/93 | 94 | 2-2 | 3-5 | 8-B1 | 13 | 2-1 | 17-1 | 17-1 | 360 | 2-6 | 11-2 | 8-B1 | 94 | 2-2 | 3-5 | 8-B1 |
| 93 | 92 | 0044/93 | 35 | 2-2 | 17-1 | 17-1 | 17 | 2-2 | 5-1 | 8-C1 | 359 | 2-2 | 5-1 | 25-1 | 13 | 2-1 | 17-1 | 17-1 |
| 94 | 92 | 0086/93 | 94 | 2-2 | 3-5 | 8-B1 | 17 | 2-2 | 5-1 | 8-C1 | 139 | 2-6 | 11-2 | 1-6 | 13 | 2-1 | 17-1 | 17-1 |
| 94 | 92 | 0224/93 | 94 | 2-2 | 3-5 | 8-B1 | 17 | 2-2 | 5-1 | 8-C1 | 139 | 2-6 | 11-2 | 1-6 | 13 | 2-1 | 17-1 | 17-1 |
| 95 | 92 | 0268/93 | 94 | 2-2 | 3-5 | 8-B1 | 60 | 2-2 | 12-1 | 4-A5 | 139 | 2-6 | 11-2 | 1-6 | 229 | 2-2 | 12-1 | 17-2 |
| 96 | 269 | 0211/93 | 101 | 2-2 | 3-5 | 19-1 | 41 | 3-3 | 15-1 | 19-1 | 153 | 2-2 | 16-2 | 19-1 | 211 | 5-2 | 16-2 | 1-6 |
| 97 | - | 0236/93 | 205 | 2-2 | 5-4 | 8-C1 | 50 | 2-2 | 10-1 | 10-4 | 157 | 2-7 | 2-2 | 1-5 | 205 | 2-2 | 5-4 | 8-C1 |
| 98 | - | 0328/93 | 125 | 2-2 | 10-1 | 10-4 | 73 | 4-3 | 10-1 | 10-4 | 170 | 2-7 | 2-1 | 1-5 | 235 | 2-2 | 5-4 | 8-C1 |
| 99 | - | 0204/93 | ININ | ININ | ININ | ININ | 38 | 4-2 | 13-1 | 16-1 | 174 | 4-2 | 13-1 | 16-1 | 222 | 4-2 | 12-1 | 4-B4 |
| 100 | - | 0229/93 | 1 | 2-1 | 1B-4 | 6-1 | 19 | 2-2 | 18-4 | 11-1 | ND | ND | ND | ND | 213 | 2-1 | 3-6 | 1-1 |
| 100 | - | 0232/93 | 1 | 2-1 | 1B-4 | 6-1 | 19 | 2-2 | 18-4 | 11-1 | ND | ND | ND | ND | 213 | 2-1 | 3-6 | 1-1 |
| 101 | - | 0105/93 | 357 | 4-2 | 12-1 | 4-B4 | 1 | 2-1 | 1B-4 | 6-1 | 1 | 2-1 | 1B-4 | 6-1 | 1 | 2-1 | 1B-4 | 6-1 |
| 101 | - | 0248/93 | 110 | 1-1 | 1-2 | 9-3 | 55 | 4-2 | 12-1 | 4-B1 | ND | ND | ND | ND | 55 | 4-2 | 12-1 | 4-B1 |
| 101 | - | 0297/93 | 55 | 4-2 | 12-1 | 4-B1 | 1 | 2-1 | 1B-4 | 6-1 | 1 | 2-1 | 1B-4 | 6-1 | 1 | 2-1 | 1B-4 | 6-1 |
| 101 | - | 0304/93 | 55 | 4-2 | 12-1 | 4-B1 | 1 | 2-1 | 1B-4 | 6-1 | 1 | 2-1 | 1B-4 | 6-1 | 1 | 2-1 | 1B-4 | 6-1 |
| 101 | - | 0336/93 | 55 | 4-2 | 12-1 | 4-B1 | 1 | 2-1 | 1B-4 | 6-1 | 1 | 2-1 | 1B-4 | 6-1 | 1 | 2-1 | 1B-4 | 6-1 |
| 102 | 18 | 0403/93 | 127 | 4-3 | 3-6 | 1-1 | 186 | 4-2 | 1A-3 | 8-2 | 143 | 2-2 | 11-2 | 1-6 | 237 | 4-7 | 15-2 | 3-1 |
| 103 | - | 0048/93 | 94 | 2-2 | 3-5 | 8-B1 | 19 | 2-2 | 18-4 | 11-1 | 98 | 5-2 | 1B-3 | 8-A4 | 55 | 4-2 | 12-1 | 4-B1 |
| 103 | - | 0116/93 | 55 | 4-2 | 12-1 | 4-B1 | 191 | 2-2 | 19-4 | 14-2 | 200 | 5-2 | 15-1 | 12-3 | ND | ND | ND | ND |
| 103 | - | 0201/93 | 98 | 5-2 | 1B-3 | 8-A4 | ININ | ININ | ININ | ININ | 98 | 5-2 | 1B-3 | 8-A4 | 157 | 2-7 | 2-2 | 1-5 |
| 104 | 549 | 0301/93 | 119 | 2-1 | 2-2 | 1-5 | 29 | 3-2 | 19-9 | 14-1 | 165 | 4-2 | 13-1 | 17-5 | 119 | 2-1 | 2-2 | 1-5 |
| 104 | 549 | 0315/93 | 24 | 4-3 | 19-10 | 11-2 | 29 | 3-2 | 19-9 | 14-1 | 165 | 4-2 | 13-1 | 17-5 | 119 | 2-1 | 2-2 | 1-5 |
| 104 | 549 | 0316/93 | 121 | 2-1 | 9-1 | 12-3 | 29 | 3-2 | 19-9 | 14-1 | 163 | 2-2 | 11-2 | 1-6 | 119 | 2-1 | 2-2 | 1-5 |
| 104 | 549 | 0319/93 | 121 | 2-1 | 9-1 | 12-3 | 29 | 3-2 | 19-9 | 14-1 | 163 | 2-2 | 11-2 | 1-6 | 119 | 2-1 | 2-2 | 1-5 |
| 105 | 549 | 0241/93 | 108 | 4-2 | 15-1 | 13-2 | 53 | 3-2 | 15-1 | 13-2 | 53 | 3-2 | 15-1 | 13-2 | 157 | 2-7 | 2-2 | 1-5 |
| 106 | 106 | 0018/93 | 138 | 5-1 | 10-5 | 3-2 | 10 | 2-2 | 19-7 | 15-2 | 138 | 5-1 | 10-5 | 3-2 | 157 | 2-7 | 2-2 | 1-5 |
| 106 | 106 | 0019/93 | 138 | 5-1 | 10-5 | 3-2 | 10 | 2-2 | 19-7 | 15-2 | 138 | 5-1 | 10-5 | 3-2 | 157 | 2-7 | 2-2 | 1-5 |
| 106 | 106 | 0020/93 | 138 | 5-1 | 10-5 | 3-2 | 10 | 2-2 | 19-7 | 15-2 | 138 | 5-1 | 10-5 | 3-2 | 157 | 2-7 | 2-2 | 1-5 |
| 106 | 106 | 0087/93 | ININ | ININ | ININ | ININ | 32 | 2-9 | 18-4 | 11-1 | 338 | 2-2 | 15-4 | 19-2 | 157 | 2-7 | 2-2 | 1-5 |
| 106 | 106 | 0097/93 | 337 | 5-1 | 10-5 | 3-5 | 13 | 2-1 | 17-1 | 17-1 | 337 | 5-1 | 10-5 | 3-5 | 157 | 2-7 | 2-2 | 1-5 |
| 106 | 106 | 0107/93 | 138 | 5-1 | 10-5 | 3-2 | 10 | 2-2 | 19-7 | 15-2 | 138 | 5-1 | 10-5 | 3-2 | 157 | 2-7 | 2-2 | 1-5 |
| 106 | 106 | 0208/93 | 152 | 5-1 | 19-A3 | 11-5 | 10 | 2-2 | 19-7 | 15-2 | 152 | 5-1 | 19-A3 | 11-5 | 157 | 2-7 | 2-2 | 1-5 |
| 106 | 106 | 0221/93 | 103 | 4-2 | 4-2 | 7-2 | 10 | 2-2 | 19-7 | 15-2 | 138 | 5-1 | 10-5 | 3-2 | 157 | 2-7 | 2-2 | 1-5 |
| 106 | 106 | 0222/93 | 138 | 5-1 | 10-5 | 3-2 | 10 | 2-2 | 19-7 | 15-2 | 138 | 5-1 | 10-5 | 3-2 | 157 | 2-7 | 2-2 | 1-5 |
| 106 | 106 | 0234/93 | 138 | 5-1 | 10-5 | 3-2 | 10 | 2-2 | 19-7 | 15-2 | 138 | 5-1 | 10-5 | 3-2 | 138 | 5-1 | 10-5 | 3-2 |
| 106 | 106 | 0245/93 | 138 | 5-1 | 10-5 | 3-2 | 10 | 2-2 | 19-7 | 15-2 | 138 | 5-1 | 10-5 | 3-2 | 157 | 2-7 | 2-2 | 1-5 |
| 106 | 106 | 0250/93 | 138 | 5-1 | 10-5 | 3-2 | 10 | 2-2 | 19-7 | 15-2 | 138 | 5-1 | 10-5 | 3-2 | 157 | 2-7 | 2-2 | 1-5 |
| 106 | 106 | 0324/93 | 138 | 5-1 | 10-5 | 3-2 | 10 | 2-2 | 19-7 | 15-2 | 138 | 5-1 | 10-5 | 3-2 | 157 | 2-7 | 2-2 | 1-5 |
| 106 | 106 | 0326/93 | 138 | 5-1 | 10-5 | 3-2 | 10 | 2-2 | 19-7 | 15-2 | 138 | 5-1 | 10-5 | 3-2 | 157 | 2-7 | 2-2 | 1-5 |
| 106 | 106 | 0382/93 | ININ | ININ | ININ | ININ | 10 | 2-2 | 19-7 | 15-2 | 138 | 5-1 | 10-5 | 3-2 | 157 | 2-7 | 2-2 | 1-5 |
| 106 | 106 | 0418/93 | 138 | 5-1 | 10-5 | 3-2 | 198 | 2-2 | 19-6 | 11-5 | 138 | 5-1 | 10-5 | 3-2 | 157 | 2-7 | 2-2 | 1-5 |
| 107 | - | 0233/93 | 105 | 4-6 | 12-1 | 4-A5 | 49 | 3-2 | 12-1 | 4-A4 | 105 | 4-6 | 12-1 | 4-A5 | 13 | 2-1 | 17-1 | 17-1 |
| 108 | 44 | 0117/93 | 98 | 5-2 | 1B-3 | 8-A4 | 14 | 2-2 | 18-1 | 15-2 | 98 | 5-2 | 1B-3 | 8-A4 | 213 | 2-1 | 3-6 | 1-1 |
| 109 | 44 | 0258/93 | 24 | 4-3 | 19-10 | 11-2 | 58 | 2-1 | 10-4 | 10-2 | 58 | 2-1 | 10-4 | 10-2 | 213 | 2-1 | 3-6 | 1-1 |
| 110 | 44 | 0067/93 | 24 | 4-3 | 19-10 | 11-2 | 14 | 2-2 | 18-1 | 15-2 | 148 | 3-2 | 1-3 | 8-A5 | 213 | 2-1 | 3-6 | 1-1 |
| 110 | 44 | 0215/93 | 24 | 4-3 | 19-10 | 11-2 | 180 | 2-2 | 18-1 | 11-2 | 148 | 3-2 | 1-3 | 8-A5 | 213 | 2-1 | 3-6 | 1-1 |
| 110 | 44 | 0225/93 | 104 | 2-2 | 19-10 | 11-2 | 14 | 2-2 | 18-1 | 15-2 | 148 | 3-2 | 1-3 | 8-A5 | 213 | 2-1 | 3-6 | 1-1 |
| 110 | 44 | 0226/93 | 104 | 2-2 | 19-10 | 11-2 | 14 | 2-2 | 18-1 | 15-2 | 148 | 3-2 | 1-3 | 8-A5 | 213 | 2-1 | 3-6 | 1-1 |
| 110 | 44 | 0227/93 | 104 | 2-2 | 19-10 | 11-2 | 14 | 2-2 | 18-1 | 15-2 | 148 | 3-2 | 1-3 | 8-A5 | 213 | 2-1 | 3-6 | 1-1 |
| 110 | 44 | 0228/93 | 104 | 2-2 | 19-10 | 11-2 | 14 | 2-2 | 18-1 | 15-2 | 148 | 3-2 | 1-3 | 8-A5 | 213 | 2-1 | 3-6 | 1-1 |
| 111 | 44 | 0216/93 | 24 | 4-3 | 19-10 | 11-2 | 44 | 2-2 | 19-10 | 15-2 | 148 | 3-2 | 1-3 | 8-A5 | 213 | 2-1 | 3-6 | 1-1 |
| 111 | 44 | 0217/93 | 24 | 4-3 | 19-10 | 11-2 | 44 | 2-2 | 19-10 | 15-2 | 148 | 3-2 | 1-3 | 8-A5 | 213 | 2-1 | 3-6 | 1-1 |
| 112 | 44 | 0047/93 | 91 | 2-2 | 19-A1 | 11-1 | 19 | 2-2 | 18-4 | 11-1 | 140 | 3-2 | 1B-6 | 8-A2 | 213 | 2-1 | 3-6 | 1-1 |
| 112 | 44 | 0091/93 | 91 | 2-2 | 19-A1 | 11-1 | 19 | 2-2 | 18-4 | 11-1 | 140 | 3-2 | 1B-6 | 8-A2 | 213 | 2-1 | 3-6 | 1-1 |
| 113 | 22 | 0061/93 | 95 | 2-1 | 1-1 | 9-3 | 19 | 2-2 | 18-4 | 11-1 | 135 | 4-3 | 16-1 | 1-2 | 217 | 2-2 | 1-1 | 9-3 |
| 114 | 22 | 0306/93 | 39 | 2-2 | 5-1 | 8-C1 | 66 | 2-2 | 18-5 | 14-A1 | 161 | 2-2 | 11-1 | 1-4 | ND | ND | ND | ND |
| 114 | 22 | 0312/93 | 39 | 2-2 | 5-1 | 8-C1 | 66 | 2-2 | 18-5 | 14-A1 | 161 | 2-2 | 11-1 | 1-4 | ND | ND | ND | ND |
| 115 | 231 | 0279/93 | 195 | 2-2 | 11-2 | 19-1 | 195 | 2-2 | 11-2 | 19-1 | 195 | 2-2 | 11-2 | 19-1 | ND | ND | ND | ND |
| 116 | 116 | 0013/93 | 88 | 2-1 | 4-2 | 7-2 | 69 | 4-2 | 9-1 | 12-3 | 145 | 4-2 | 12-2 | 21-1 | 88 | 2-1 | 4-2 | 7-2 |
| 116 | 116 | 0014/93 | 203 | 2-1 | 4-2 | 6-1 | 69 | 4-2 | 9-1 | 12-3 | 88 | 2-1 | 4-2 | 7-2 | 88 | 2-1 | 4-2 | 7-2 |
| 116 | 116 | 0078/93 | 88 | 2-1 | 4-2 | 7-2 | 28 | 4-4 | 9-1 | 12-3 | 88 | 2-1 | 4-2 | 7-2 | 88 | 2-1 | 4-2 | 7-2 |
| 116 | 116 | 0203/93 | 88 | 2-1 | 4-2 | 7-2 | 37 | 4-2 | 4-2 | 7-2 | 88 | 2-1 | 4-2 | 7-2 | 88 | 2-1 | 4-2 | 7-2 |
| 116 | 116 | 0219/93 | 88 | 2-1 | 4-2 | 7-2 | 45 | 4-5 | 17-1 | 17-1 | 88 | 2-1 | 4-2 | 7-2 | ND | ND | ND | ND |
| 116 | 116 | 0223/93 | 103 | 4-2 | 4-2 | 7-2 | 46 | 2-2 | 7-1 | 5-5 | 158 | 4-7 | 15-2 | 1-7 | 88 | 2-1 | 4-2 | 7-2 |
| 116 | 116 | 0240/93 | 88 | 2-1 | 4-2 | 7-2 | 45 | 4-5 | 17-1 | 17-1 | 88 | 2-1 | 4-2 | 7-2 | ND | ND | ND | ND |
| 116 | 116 | 0308/93 | 1 | 2-1 | 1B-4 | 6-1 | 67 | 4-2 | 9-2 | 12-3 | 88 | 2-1 | 4-2 | 7-2 | 88 | 2-1 | 4-2 | 7-2 |
| 116 | 116 | 0321/93 | 123 | 2-1 | 1B-5 | 6-1 | 69 | 4-2 | 9-1 | 12-3 | 123 | 2-1 | 1B-5 | 6-1 | 88 | 2-1 | 4-2 | 7-2 |
| 116 | 116 | 0401/93 | 88 | 2-1 | 4-2 | 7-2 | 76 | 2-1 | 4-2 | 12-3 | 145 | 4-2 | 12-2 | 21-1 | 88 | 2-1 | 4-2 | 7-2 |
| 116 | 116 | 0406/93 | 88 | 2-1 | 4-2 | 7-2 | 76 | 2-1 | 4-2 | 12-3 | 145 | 4-2 | 12-2 | 21-1 | 88 | 2-1 | 4-2 | 7-2 |
| 116 | 116 | 0413/93 | 88 | 2-1 | 4-2 | 7-2 | 76 | 2-1 | 4-2 | 12-3 | 145 | 4-2 | 12-2 | 21-1 | 88 | 2-1 | 4-2 | 7-2 |
| 117 | - | 0038/93 | 350 | 4-2 | 6-1 | 9-2 | 13 | 2-1 | 17-1 | 17-1 | 162 | 2-2 | 13-1 | 16-2 | 220 | 2-2 | 19-1 | 4-3 |
| 117 | - | 0040/93 | 350 | 4-2 | 6-1 | 9-2 | 16 | 2-1 | 17-1 | 17-1 | 162 | 2-2 | 13-1 | 16-2 | 220 | 2-2 | 19-1 | 4-3 |
| 117 | - | 0077/93 | 350 | 4-2 | 6-1 | 9-2 | 27 | 4-2 | 7-1 | 5-2 | 162 | 2-2 | 13-1 | 16-2 | 220 | 2-2 | 19-1 | 4-3 |
| 117 | - | 0267/93 | 115 | 4-2 | 13-1 | 16-2 | 27 | 4-2 | 7-1 | 5-2 | 162 | 2-2 | 13-1 | 16-2 | 220 | 2-2 | 19-1 | 4-3 |
| 118 | 32 | 0062/93 | 96 | 2-1 | 19-10 | 14-3 | 185 | 5-1 | 10-5 | 3-1 | 147 | 2-2 | 1A-2 | 8-1 | 218 | 2-1 | 19-10 | 14-5 |
| 119 | 106 | 0070/93 | 157 | 2-7 | 2-2 | 1-5 | 26 | 2-2 | 10-1 | 10-3 | 157 | 2-7 | 2-2 | 1-5 | 157 | 2-7 | 2-2 | 1-5 |
| 120 | - | 0237/93 | 106 | 4-2 | 12-1 | 4-A6 | 46 | 2-2 | 7-1 | 5-5 | 158 | 4-7 | 15-2 | 1-7 | 226 | 3-2 | 19-1 | 4-3 |
| 121 | - | 0057/93 | 93 | 4-2 | 1A-1 | 8-A1 | 23 | 2-1 | 1B-2 | 5-7 | 145 | 4-2 | 12-2 | 21-1 | 157 | 2-7 | 2-2 | 1-5 |
| 121 | - | 0058/93 | 93 | 4-2 | 1A-1 | 8-A1 | 23 | 2-1 | 1B-2 | 5-7 | 145 | 4-2 | 12-2 | 21-1 | 157 | 2-7 | 2-2 | 1-5 |
| 122 | 53 | 0206/93 | 183 | 2-1 | 17-2 | 17-6 | 25 | 2-3 | 19-1 | 2-1 | 25 | 2-3 | 19-1 | 2-1 | 132 | 2-2 | 11-2 | 1-6 |
| 123 | 53 | 0277/93 | 55 | 4-2 | 12-1 | 4-B1 | 61 | 4-2 | 1B-4 | 6-1 | ND | ND | ND | ND | 230 | 2-2 | 11-2 | 1-6 |
| 124 | 53 | 0314/93 | 120 | 2-2 | 11-2 | 17-4 | 25 | 2-3 | 19-1 | 2-1 | ND | ND | ND | ND | 132 | 2-2 | 11-2 | 1-6 |
| 125 | - | 0055/93 | 92 | 2-5 | 9-1 | 12-3 | 22 | 2-2 | 3-5 | 9-A1 | 144 | 2-2 | 9-1 | 9-A1 | 216 | 3-2 | 2-2 | 1-5 |
| 125 | - | 0056/93 | 92 | 2-5 | 9-1 | 12-3 | 22 | 2-2 | 3-5 | 9-A1 | 144 | 2-2 | 9-1 | 9-A1 | 216 | 3-2 | 2-2 | 1-5 |
| 125 | - | 0106/93 | 92 | 2-5 | 9-1 | 12-3 | 22 | 2-2 | 3-5 | 9-A1 | 144 | 2-2 | 9-1 | 9-A1 | 216 | 3-2 | 2-2 | 1-5 |
| 125 | - | 0108/93 | 92 | 2-5 | 9-1 | 12-3 | 22 | 2-2 | 3-5 | 9-A1 | 144 | 2-2 | 9-1 | 9-A1 | 216 | 3-2 | 2-2 | 1-5 |
| 125 | - | 0299/93 | 118 | 2-2 | 18-6 | 14-1 | 64 | 2-1 | 9-1 | 12-3 | 164 | 2-2 | 3-5 | 9-A1 | 216 | 3-2 | 2-2 | 1-5 |
| 125 | - | 0300/93 | ND | ND | ND | ND | 22 | 2-2 | 3-5 | 9-A1 | 164 | 2-2 | 3-5 | 9-A1 | 216 | 3-2 | 2-2 | 1-5 |
| 126 | - | 0015/93 | 646 | 2-2 | 18-4 | 11-2 | 178 | 2-1 | 17-1 | 17-1 | 137 | 2-2 | 10-3 | 5-8 | 646 | 2-2 | 18-4 | 11-2 |
| 127 | - | 0029/93 | 12 | 4-2 | 6-1 | 9-2 | 12 | 4-2 | 6-1 | 9-2 | 12 | 4-2 | 6-1 | 9-2 | 12 | 4-2 | 6-1 | 9-2 |
| 127 | - | 0063/93 | 12 | 4-2 | 6-1 | 9-2 | 12 | 4-2 | 6-1 | 9-2 | 12 | 4-2 | 6-1 | 9-2 | 12 | 4-2 | 6-1 | 9-2 |
| 127 | - | 0210/93 | 12 | 4-2 | 6-1 | 9-2 | 12 | 4-2 | 6-1 | 9-2 | 12 | 4-2 | 6-1 | 9-2 | 12 | 4-2 | 6-1 | 9-2 |
| 128 | - | 0254/93 | 112 | 4-2 | 5-1 | 8-C1 | 3 | 2-2 | 10-1 | 10-3 | 3 | 2-2 | 10-1 | 10-3 | ND | ND | ND | ND |
| 129 | 92 | 0068/93 | 210 | 2-2 | 17-1 | 17-1 | 13 | 2-1 | 17-1 | 17-1 | 139 | 2-6 | 11-2 | 1-6 | 13 | 2-1 | 17-1 | 17-1 |
| 130 | - | 0017/93 | 90 | 3-2 | 13-2 | 16-1 | 9 | 2-2 | 1A-1 | 18-1 | 90 | 3-2 | 13-2 | 16-1 | 90 | 3-2 | 13-2 | 16-1 |
| 130 | - | 0111/93 | 638 | 2-1 | 19-7 | 4-1 | 197 | 2-1 | 1A-1 | 8-A1 | 157 | 2-7 | 2-2 | 1-5 | ND | ND | ND | ND |
| 130 | - | 0256/93 | 90 | 3-2 | 13-2 | 16-1 | 182 | 4-2 | 14-2 | 4-A1 | 90 | 3-2 | 13-2 | 16-1 | ND | ND | ND | ND |
| 131 | - | 0212/93 | 101 | 2-2 | 3-5 | 19-1 | 42 | 4-2 | 6-1 | 9-2 | 154 | 2-2 | 19-10 | 11-2 | 87 | 2-2 | 11-1 | 1-4 |
| 132 | - | 0239/93 | 640 | 2-1 | 10-6 | 10-9 | 52 | 4-2 | 5-1 | 8-C1 | 160 | 2-8 | 19-10 | 11-2 | 87 | 2-2 | 11-1 | 1-4 |
| 132 | - | 0335/93 | 640 | 2-1 | 10-6 | 10-9 | 52 | 4-2 | 5-1 | 8-C1 | 160 | 2-8 | 19-10 | 11-2 | 87 | 2-2 | 11-1 | 1-4 |
| 132 | - | 0337/93 | 640 | 2-1 | 10-6 | 10-9 | 52 | 4-2 | 5-1 | 8-C1 | 160 | 2-8 | 19-10 | 11-2 | 87 | 2-2 | 11-1 | 1-4 |
| 133 | 116 | 0327/93 | 641 | 2-8 | 1A-4 | 12-1 | 72 | 4-2 | 19-3 | 14-1 | 169 | 2-2 | 1A-4 | 12-1 | 234 | 3-2 | 18-2 | 16-4 |
| 134 | - | 0231/93 | 14 | 2-2 | 18-1 | 15-2 | 48 | 2-2 | 1-1 | 9-3 | 156 | 2-1 | 7-1 | 5-3 | 225 | 4-3 | 12A-1 | 1-1 |
| 135 | - | 0257/93 | 114 | 4-4 | 7-1 | 5-2 | 57 | 2-2 | 11-1 | 1-3 | 57 | 2-2 | 11-1 | 1-3 | ND | ND | ND | ND |
| 136 | 44 | 0325/93 | 71 | 4-2 | 19-7 | 4-1 | 71 | 4-2 | 19-7 | 4-1 | 168 | 4-2 | 6-1 | 8-C1 | 236 | 2-2 | 9-3 | 3-4 |
| 136 | 44 | 0342/93 | 71 | 4-2 | 19-7 | 4-1 | 71 | 4-2 | 19-7 | 4-1 | 168 | 4-2 | 6-1 | 8-C1 | 236 | 2-2 | 9-3 | 3-4 |
| 136 | 44 | 0404/93 | 71 | 4-2 | 19-7 | 4-1 | 71 | 4-2 | 19-7 | 4-1 | 168 | 4-2 | 6-1 | 8-C1 | 238 | 4-2 | 9-3 | 3-4 |
| 136 | 44 | 0412/93 | 71 | 4-2 | 19-7 | 4-1 | 71 | 4-2 | 19-7 | 4-1 | 168 | 4-2 | 6-1 | 8-C1 | 238 | 4-2 | 9-3 | 3-4 |
| 136 | 44 | 0414/93 | 71 | 4-2 | 19-7 | 4-1 | 71 | 4-2 | 19-7 | 4-1 | 168 | 4-2 | 6-1 | 8-C1 | 238 | 4-2 | 9-3 | 3-4 |
| 136 | 44 | 0421/93 | 71 | 4-2 | 19-7 | 4-1 | 71 | 4-2 | 19-7 | 4-1 | 168 | 4-2 | 6-1 | 8-C1 | 238 | 4-2 | 9-3 | 3-4 |
| 137 | 44 | 0280/93 | 154 | 2-2 | 19-10 | 11-2 | 62 | 3-2 | 12-1 | 15-4 | ND | ND | ND | ND | 213 | 2-1 | 3-6 | 1-1 |
| 138 | - | 0322/93 | 124 | 2-2 | 19-1 | 15-2 | 70 | 2-2 | 16-2 | 3-1 | 167 | 2-2 | 7-1 | 3-3 | 233 | 4-3 | 16-2 | 1-1 |
| 139 | 231 | 0339/93 | 14 | 2-2 | 18-1 | 15-2 | 53 | 3-2 | 15-1 | 13-2 | 139 | 2-6 | 11-2 | 1-6 | 135 | 4-3 | 16-1 | 1-2 |
| 140 | - | 0341/93 | 12 | 4-2 | 6-1 | 9-2 | 12 | 4-2 | 6-1 | 9-2 | 12 | 4-2 | 6-1 | 9-2 | 12 | 4-2 | 6-1 | 9-2 |
| 141 | 292 | 0405/93 | 202 | 4-2 | 4-3 | 9-1 | 187 | 2-1 | 18-1 | 15-2 | ND | ND | ND | ND | 239 | 2-1 | 4-4 | 1-1 |
| 142 | 44 | 0419/93 | 24 | 4-3 | 19-10 | 11-2 | ININ | ININ | ININ | ININ | 176 | 2-2 | 5-3 | 6-A1 | 213 | 2-1 | 3-6 | 1-1 |
| 143 | - | 0423/93 | 209 | 2-2 | 1-1 | 9-3 | ND | ND | ND | ND | 177 | 2-2 | 5-2 | 8-C1 | 127 | 4-3 | 3-6 | 1-1 |
| 144 | - | 0425/93 | 55 | 4-2 | 12-1 | 4-B1 | 361 | 2-1 | 1-B8 | 9-A2 | 55 | 4-2 | 12-1 | 4-B1 | 241 | 4-2 | 11-3 | 1-4 |
| 145 | 18 | 0427/93 | 87 | 2-2 | 11-1 | 1-4 | 5 | 2-2 | 7-1 | 5-4 | 5 | 2-2 | 7-1 | 5-4 | 87 | 2-2 | 11-1 | 1-4 |
| 215 | - | 0397/93 | 126 | 2-2 | 3-1 | 8-B1 | 75 | 4-6 | 8-1 | 9-3 | 171 | 2-2 | 19-7 | 14-4 | 89 | 2-2 | 19-7 | 4-B2 |
| 216 | 106 | 0407/93 | 138 | 5-1 | 10-5 | 3-2 | 10 | 2-2 | 19-7 | 15-2 | 138 | 5-1 | 10-5 | 3-2 | 157 | 2-7 | 2-2 | 1-5 |
| 670 | - | 0271/93 | 128 | 2-2 | 3-5 | 6-1 | 193 | 3-2 | 13-2 | 16-3 | 201 | 2-2 | 4-1 | 7-3 | ND | ND | ND | ND |
| 672 | 11 | 0104/93 | 83 | 3-4 | 5-2 | 18-1 | 11 | 3-1 | 18-3 | 14-1 | 132 | 2-2 | 11-2 | 1-6 | ININ | ININ | ININ | ININ |
| 690 | 44 | 0409/93 | 358 | 2-2 | 18-8 | 11-3 | 36 | 2-1 | 7-1 | 5-2 | 36 | 2-1 | 7-1 | 5-2 | 213 | 2-1 | 3-6 | 1-1 |
| 709 | - | 0112/93 | 90 | 3-2 | 13-2 | 16-1 | 90 | 3-2 | 13-2 | 16-1 | 98 | 5-2 | 1B-3 | 8-A4 | 90 | 3-2 | 13-2 | 16-1 |
| 721 | 44 | 0120/93 | 24 | 4-3 | 19-10 | 11-2 | ND | ND | ND | ND | 14 | 2-2 | 18-1 | 15-2 | ND | ND | ND | ND |
| 726 | - | 0034/93 | 15 | 4-2 | 19-8 | 14-1 | 15 | 4-2 | 19-8 | 14-1 | 351 | 4-2 | 6-1 | 9-2 | 351 | 4-2 | 6-1 | 9-2 |
| 733 | 11 | 0033/93 | 83 | 3-4 | 5-2 | 18-1 | 11 | 3-1 | 18-3 | 14-1 | ND | ND | ND | ND | ININ | ININ | ININ | ININ |
| 734 | 92 | 0110/93 | 94 | 2-2 | 3-5 | 8-B1 | 190 | 2-2 | 3-5 | 17-1 | 139 | 2-6 | 11-2 | 1-6 | 94 | 2-2 | 3-5 | 8-B1 |
| 742 | - | 0305/93 | 208 | 2-2 | 3-2 | 8-A3 | 183 | 2-1 | 17-2 | 17-6 | 158 | 4-7 | 15-2 | 1-7 | 208 | 2-2 | 3-2 | 8-A3 |
| 743 | - | 0031/93 | 285 | 2-1 | 19-3 | 2-1 | 14 | 2-2 | 18-1 | 15-2 | 336 | 3-2 | 2-2 | 1-11 | 216 | 3-2 | 2-2 | 1-5 |
| 989 | 18 | 0118/93 | 2 | 4-1 | 6-1 | 9-2 | 192 | 4-1 | 1A-4 | 12-1 | 2 | 4-1 | 6-1 | 9-2 | 2 | 4-1 | 6-1 | 9-2 |
| 1000 | 35 | 0060/93 | 265 | 3-2 | 7-1 | 5-9 | ND | ND | ND | ND | 265 | 3-2 | 7-1 | 5-9 | 211 | 5-2 | 16-2 | 1-6 |
| 1002 | - | 0084/93 | 356 | 4-2 | 12-1 | 4-B6 | 31 | 4-3 | 19-10 | 11-2 | 98 | 5-2 | 1B-3 | 8-A4 | ND | ND | ND | ND |
| 1004 | - | 0088/93 | 356 | 4-2 | 12-1 | 4-B6 | 31 | 4-3 | 19-10 | 11-2 | 98 | 5-2 | 1B-3 | 8-A4 | ND | ND | ND | ND |
| 1086 | 44 | 0071/93 | 355 | 4-2 | 12-1 | 4-B1 | 14 | 2-2 | 18-1 | 15-2 | 113 | 2-2 | 10-1 | 10-3 | 127 | 4-3 | 3-6 | 1-1 |
| 1087 | 106 | 0081/93 | ND | ND | ND | ND | 29 | 3-2 | 19-9 | 14-1 | 138 | 5-1 | 10-5 | 3-2 | 157 | 2-7 | 2-2 | 1-5 |
| ? | ? | 0552-93 | 2 | 4-1 | 6-1 | 9-2 | 63 | 4-3 | 18-3 | 11-2 | 2 | 4-1 | 6-1 | 9-2 | 2 | 4-1 | 6-1 | 9-2 |
| ? | ? | 0075-93 | ND | ND | ND | ND | ININ | ININ | ININ | ININ | 132 | 2-2 | 11-2 | 1-6 | ND | ND | ND | ND |
| ? | ? | 0540-93 | 83 | 3-4 | 5-2 | 18-1 | 11 | 3-1 | 18-3 | 14-1 | 132 | 2-2 | 11-2 | 1-6 | ININ | ININ | ININ | ININ |
