# Supplementary material for: The Effect of Immune Selection on the Structure of the Meningococcal Opa Protein Repertoire
Source: PLoS Pathog. 2008 Mar 14;4(3):e1000020. doi: 10.1371/journal.ppat.1000020 (PMC2265424; doi:10.1371/journal.ppat.1000020)
Supplement: Table S2 — opa repertoires of meningococci isolated from invasive disease in the Czech Republic during 1993. ST: multilocus sequence typing (MLST) sequence type, CC: MLST clonal complex SV: semi variable region variant, HV1: first hypervariable region variant, HV2: second hypervariable region variant, ND: opa sequence not detected, ININ: insertional inactivation of opa locus by insertion sequence-like element, FSM: opa locus present but non-functional due to frameshift mutation. (0.19 MB DOC) [file ppat.1000020.s002.doc]

**Supplementary Table 2.** *opa* repertoires of meningococci isolated from invasive disease in the Czech Republic during 1993. ST: multilocus sequence typing (MLST) sequence type, CC: MLST clonal complex SV: semi variable region variant, HV1: first hypervariable region variant, HV2: second hypervariable region variant, ND: opa sequence not detected, ININ: insertional inactivation of *opa* locus by insertion sequence-like element, FSM: opa locus present but non-functional due to frameshift mutation.

| **ST** | **CC** | **Isolate** | **opaA** | **Sv** | **Hv1** | **Hv2** | **opaB** | **Sv** | **Hv1** | **Hv2** | **opaD** | **Sv** | **Hv1** | **Hv2** | **opaJ** | **Sv** | **Hv1** | **Hv2** |
| --- | --- | --- | --- | --- | --- | --- | --- | --- | --- | --- | --- | --- | --- | --- | --- | --- | --- | --- |
| 11 | 11 | 0002/93 | 83 | 3-4 | 5-2 | 18-1 | 11 | 3-1 | 18-3 | 14-1 | 132 | 2-2 | 11-2 | 1-6 | ININ | ININ | ININ | ININ |
| 11 | 11 | 0186/93 | 83 | 3-4 | 5-2 | 18-1 | ININ | ININ | ININ | ININ | 132 | 2-2 | 11-2 | 1-6 | ININ | ININ | ININ | ININ |
| 11 | 11 | 0188/93 | 83 | 3-4 | 5-2 | 18-1 | 34 | 4-3 | 18-3 | 14-1 | 132 | 2-2 | 11-2 | 1-6 | ININ | ININ | ININ | ININ |
| 11 | 11 | 0259/93 | 83 | 3-4 | 5-2 | 18-1 | 11 | 3-1 | 18-3 | 14-1 | 132 | 2-2 | 11-2 | 1-6 | ININ | ININ | ININ | ININ |
| 11 | 11 | 0263/93 | 83 | 3-4 | 5-2 | 18-1 | 11 | 3-1 | 18-3 | 14-1 | 132 | 2-2 | 11-2 | 1-6 | ININ | ININ | ININ | ININ |
| 11 | 11 | 0264/93 | 83 | 3-4 | 5-2 | 18-1 | 11 | 3-1 | 18-3 | 14-1 | 132 | 2-2 | 11-2 | 1-6 | ININ | ININ | ININ | ININ |
| 11 | 11 | 0284/93 | 83 | 3-4 | 5-2 | 18-1 | 11 | 3-1 | 18-3 | 14-1 | 132 | 2-2 | 11-2 | 1-6 | ININ | ININ | ININ | ININ |
| 11 | 11 | 0287/93 | 83 | 3-4 | 5-2 | 18-1 | 11 | 3-1 | 18-3 | 14-1 | 132 | 2-2 | 11-2 | 1-6 | ININ | ININ | ININ | ININ |
| 11 | 11 | 0343/93 | 83 | 3-4 | 5-2 | 18-1 | 11 | 3-1 | 18-3 | 14-1 | 132 | 2-2 | 11-2 | 1-6 | ININ | ININ | ININ | ININ |
| 11 | 11 | 0351/93 | 83 | 3-4 | 5-2 | 18-1 | 11 | 3-1 | 18-3 | 14-1 | 132 | 2-2 | 11-2 | 1-6 | ININ | ININ | ININ | ININ |
| 11 | 11 | 0393/93 | 83 | 3-4 | 5-2 | 18-1 | 11 | 3-1 | 18-3 | 14-1 | 132 | 2-2 | 11-2 | 1-6 | ININ | ININ | ININ | ININ |
| 11 | 11 | 0400/93 | 83 | 3-4 | 5-2 | 18-1 | 11 | 3-1 | 18-3 | 14-1 | 132 | 2-2 | 11-2 | 1-6 | ININ | ININ | ININ | ININ |
| 11 | 11 | 0489/93 | 83 | 3-4 | 5-2 | 18-1 | 11 | 3-1 | 18-3 | 14-1 | 132 | 2-2 | 11-2 | 1-6 | ININ | ININ | ININ | ININ |
| 11 | 11 | 0490/93 | 83 | 3-4 | 5-2 | 18-1 | 11 | 3-1 | 18-3 | 14-1 | 132 | 2-2 | 11-2 | 1-6 | ININ | ININ | ININ | ININ |
| 11 | 11 | 0500/93 | 83 | 3-4 | 5-2 | 18-1 | 11 | 3-1 | 18-3 | 14-1 | 132 | 2-2 | 11-2 | 1-6 | ININ | ININ | ININ | ININ |
| 11 | 11 | 0512/93 | 83 | 3-4 | 5-2 | 18-1 | 11 | 3-1 | 18-3 | 14-1 | 132 | 2-2 | 11-2 | 1-6 | ININ | ININ | ININ | ININ |
| 11 | 11 | 0520/93 | 83 | 3-4 | 5-2 | 18-1 | 189 | 3-4 | 5-2 | 14-1 | 132 | 2-2 | 11-2 | 1-6 | ININ | ININ | ININ | ININ |
| 11 | 11 | 0529/93 | 83 | 3-4 | 5-2 | 18-1 | 11 | 3-1 | 18-3 | 14-1 | 132 | 2-2 | 11-2 | 1-6 | ININ | ININ | ININ | ININ |
| 11 | 11 | 0535/93 | 83 | 3-4 | 5-2 | 18-1 | 80 | 3-4 | 18-3 | 14-1 | 132 | 2-2 | 11-2 | 1-6 | ININ | ININ | ININ | ININ |
| 11 | 11 | 0538/93 | 83 | 3-4 | 5-2 | 18-1 | 11 | 3-1 | 18-3 | 14-1 | 132 | 2-2 | 11-2 | 1-6 | ININ | ININ | ININ | ININ |
| 18 | 18 | 0006/93 | 85 | 2-2 | 19-7 | 4-1 | 2 | 4-1 | 6-1 | 9-2 | 85 | 2-2 | 19-7 | 4-1 | 87 | 2-2 | 11-1 | 1-4 |
| 36 | - | 0282/93 | 116 | 4-2 | 19-A2 | 11-5 | 63 | 4-3 | 18-3 | 11-2 | 135 | 4-3 | 16-1 | 1-2 | ND | ND | ND | ND |
| 36 | - | 0283/93 | 116 | 4-2 | 19-A2 | 11-5 | 63 | 4-3 | 18-3 | 11-2 | 135 | 4-3 | 16-1 | 1-2 | ND | ND | ND | ND |
| 86 | - | 0012/93 | 7 | 4-2 | 10-2 | 10-4 | 7 | 4-2 | 10-2 | 10-4 | 135 | 4-3 | 16-1 | 1-2 | 212 | 4-6 | 12A-1 | 20-1 |
| 101 | - | 0390/93 | 55 | 4-2 | 12-1 | 4-B1 | 55 | 4-2 | 12-1 | 4-B1 | ND | ND | ND | ND | 1 | 2-1 | 1B-4 | 6-1 |
| 117 | - | 0503/93 | 350 | 4-2 | 6-1 | 9-2 | 27 | 4-2 | 7-1 | 5-2 | 162 | 2-2 | 13-1 | 16-2 | 220 | 2-2 | 19-1 | 4-3 |
| 118 | 32 | 0379/93 | 96 | 2-1 | 19-10 | 14-3 | 185 | 5-1 | 10-5 | 3-1 | 147 | 2-2 | 1A-2 | 8-1 | 218 | 2-1 | 19-10 | 14-5 |
| 118 | 32 | 0469/93 | 129 | 2-1 | 19-10 | 14-3 | 77 | 2-2 | 1B-4 | 6-1 | 147 | 2-2 | 1A-2 | 8-1 | 218 | 2-1 | 19-10 | 14-5 |
| 145 | 18 | 0010/93 | 87 | 2-2 | 11-1 | 1-4 | 5 | 2-2 | 7-1 | 5-4 | 136 | 2-2 | 19-5 | 2-1 | 87 | 2-2 | 11-1 | 1-4 |
| 145 | 18 | 0177/93 | 350 | 4-2 | 6-1 | 9-2 | 33 | 2-1 | 19-10 | 11-3 | 149 | 4-2 | 12-1 | 4-B3 | 87 | 2-2 | 11-1 | 1-4 |
| 231 | 231 | 0009/93 | 86 | 4-2 | 12-1 | 4-B1 | 4 | 4-2 | 1-1 | 12-1 | 135 | 4-3 | 16-1 | 1-2 | 135 | 4-3 | 16-1 | 1-2 |
| 668 | - | 0001/93 | ND | ND | ND | ND | 637 | 2-1 | 8-2 | 9A-5 | 173 | 3-2 | 16-2 | 1-6 | 211 | 5-2 | 16-2 | 1-6 |
| 669 | - | 0184/93 | 99 | 3-2 | 4-1 | 7-3 | 99 | 3-2 | 4-1 | 7-3 | 99 | 3-2 | 4-1 | 7-3 | 211 | 5-2 | 16-2 | 1-6 |
| 669 | - | 0443/93 | 128 | 2-2 | 3-5 | 6-1 | 188 | 2-2 | 18-6 | 11-6 | 99 | 3-2 | 4-1 | 7-3 | 211 | 5-2 | 16-2 | 1-6 |
| 678 | - | 0003/93 | 55 | 4-2 | 12-1 | 4-B1 | 1 | 2-1 | 1B-4 | 6-1 | 1 | 2-1 | 1B-4 | 6-1 | 1 | 2-1 | 1B-4 | 6-1 |
| 678 | - | 0181/93 | 55 | 4-2 | 12-1 | 4-B1 | 1 | 2-1 | 1B-4 | 6-1 | 1 | 2-1 | 1B-4 | 6-1 | 1 | 2-1 | 1B-4 | 6-1 |
| 698 | - | 0347/93 | 71 | 4-2 | 19-7 | 4-1 | 74 | 2-2 | 19-7 | 4-1 | 135 | 4-3 | 16-1 | 1-2 | ND | ND | ND | ND |
| 698 | - | 0483/93 | 71 | 4-2 | 19-7 | 4-1 | 71 | 4-2 | 19-7 | 4-1 | 135 | 4-3 | 16-1 | 1-2 | ND | ND | ND | ND |
| 704 | - | 0517/93 | 131 | 4-6 | 13-1 | 16-1 | 79 | 2-2 | 7-1 | 5-2 | 172 | 2-7 | 3-3 | 9-A1 | 211 | 5-2 | 16-2 | 1-6 |
| 718 | - | 0007/93 | 1 | 2-1 | 1B-4 | 6-1 | 3 | 2-2 | 10-1 | 10-3 | 134 | 4-2 | 11-2 | 1-6 | 352 | 2-2 | 5-1 | 11-2 |
| 720 | 18 | 0350/93 | 350 | 4-2 | 6-1 | 9-2 | 33 | 2-1 | 19-10 | 11-3 | 162 | 2-2 | 13-1 | 16-2 | 87 | 2-2 | 11-1 | 1-4 |
| 732 | - | 0050/93 | 1 | 2-1 | 1B-4 | 6-1 | 1 | 2-1 | 1B-4 | 6-1 | 142 | 4-6 | 12A-1 | 20-1 | 135 | 4-3 | 16-1 | 1-2 |
| 737 | 18 | 0049/93 | 20 | 3-2 | 4-1 | 7-1 | 20 | 3-2 | 4-1 | 7-1 | 141 | 2-2 | 1A-3 | 8-1 | 214 | 2-2 | 11-1 | 1-6 |
| 740 | 44 | 0011/93 | 24 | 4-3 | 19-10 | 11-2 | 6 | 2-2 | 10-3 | 10-1 | 6 | 2-2 | 10-3 | 10-1 | 213 | 2-1 | 3-6 | 1-1 |
| 742 | - | 0497/93 | 642 | 2-2 | 10-5 | 3-1 | 196 | 2-1 | 10-5 | 3-1 | 158 | 4-7 | 15-2 | 1-7 | 643 | 5-2 | 3-11 | 8A-3 |
| 988 | 18 | 0539/93 | 645 | 4-1 | 1A-1 | 8A-1 | 81 | 2-1 | 7-3 | 5-4 | FSM | FSM | FSM | FSM | 87 | 2-2 | 11-1 | 1-4 |
| 994 | 292 | 0502/93 | 130 | 3-2 | 12-1 | 20-1 | 78 | 2-1 | 1A-3 | 8-1 | 130 | 3-2 | 12-1 | 20-1 | 94 | 2-2 | 3-5 | 8-B1 |
| 996 | - | 0192/93 | 151 | 4-2 | 18-3 | 14-1 | 179 | 2-2 | 19-7 | 14-1 | 151 | 4-2 | 18-3 | 14-1 | 639 | 4-2 | 10-4 | 10-2 |
| 998 | 269 | 0190/93 | 206 | 2-1 | 1-1 | 8-A4 | 21 | 2-1 | 18-2 | 15-3 | 150 | 2-1 | 1-1 | 5-6 | 211 | 5-2 | 16-2 | 1-6 |
| 1001 | 18 | 0553/93 | 350 | 4-2 | 6-1 | 9-2 | 11 | 3-1 | 18-3 | 14-1 | 166 | 2-2 | 19-3 | 2-1 | 87 | 2-2 | 11-1 | 1-4 |
| 1015 | 32 | 0531/93 | 96 | 2-1 | 19-10 | 14-3 | 199 | 2-2 | 6-1 | 9-2 | 147 | 2-2 | 1A-2 | 8-1 | 218 | 2-1 | 19-10 | 14-5 |
| 1019 | - | 0005/93 | 84 | 2-4 | 19-4 | 14-2 | 1 | 2-1 | 1B-4 | 6-1 | 133 | 2-2 | 11-1 | 1-4 | 14 | 2-2 | 18-1 | 15-2 |
| 1088 | 18 | 0554/93 | 644 | 4-2 | 3-5 | 8B-1 | 82 | 2-2 | 7-2 | 5-4 | 82 | 2-2 | 7-2 | 5-4 | 87 | 2-2 | 11-1 | 1-4 |
