# Supplementary material for: The Effect of Immune Selection on the Structure of the Meningococcal Opa Protein Repertoire
Source: PLoS Pathog. 2008 Mar 14;4(3):e1000020. doi: 10.1371/journal.ppat.1000020 (PMC2265424; doi:10.1371/journal.ppat.1000020)
Supplement: Text S1 — Supplementary material (0.10 MB DOC) [file ppat.1000020.s004.doc]

**Supplementary Online Material**

1. *Diversity and structuring of the opa gene repertoire in a carried meningococcal population*

The four known *opa* loci were analysed in the 216 meningococcal isolates from a carried population sample: a total of 864 loci. In 784 loci (90.74%) an intact *opa* sequence was detected, 41 loci (4.75%) were disrupted by an IS1301-like insertion sequence, 1 locus (0.12%) was disrupted by a frameshift mutation and a sequence could not be determined at 38 loci (4.39%). A full length *opa* allele could be detected at the *opaA* locus in a total of 209/216 isolates (96.76%), in the *opaB* locus of 206/216 isolates (95.372%), in the *opaD* locus of 203/216 isolate (93.98%) at and in the *opaJ* locus of 166/216 isolates (76.85%) A further 31 isolates had an insertionally inactivated *opaJ* locus. The 784 loci contained a total of 222 alleles (nucleotide *p* distance: 13.59%). These encoded 22 SV variants (amino acid *p* distance: 33.6%) which fell into 5 families; 76 HV1 variants (amino acid *p* distance: 47.8%) which fell into 19 families and 93 HV2 variants (amino acid *p* distance: 37.6%) which fell into 21 families. Figure 1 shows the frequencies of the combination of HV1-HV2 epitopes as a bar chart (the heatmap equivalent is shown in Figure 3 of the main text).

*Figure 1: The combination of HV1-HV2 epitopes occurring in carried isolates. The height of the bars corresponds to the total number of observations of each particular combination.*

*.*

A total of 212 *opa* loci were also analysed in a contemporaneous collection of 53 isolates from invasive disease. In 185 loci (87.26%) an *opa* sequence was detected, 21 loci (9.91%) were disrupted by an IS1301-like insertion sequence and the sequence at 6 loci (2.83%) could not be determined. The 212 loci contained a total of 75 alleles (nucleotide *p* distance: 14.26%). These encoded 14 SV variants (amino acid *p* distance: 33.8%) which fell into 4 families; 41 HV1 variants (amino acid *p* distance: 48.4%) which fell into 15 families and 44 HV2 variants (amino acid *p* distance: 40.1%) which fell into 17 families.

Overlap in the repertoires of different clonal complexes was apparent. For example, the most common HV1/HV2 combination was HV1:11-2, HV2: 1-6, which was present in 8.29% (65 out of 784) of loci containing a full length *opa* allele in the data set. Of these, 31 were found in the ST-11 complex, 1 in the ST-18 complex, 8 in the ST-53 complex, 18 in the ST-92 complex, 1 in the ST-231 complex, 2 in the ST-549 complex and 4 in isolates unassigned to clonal complexes. Structuring of the diversity in individual MSLT sequences types and clonal complexes was observed. As examples, the repertoires of a number of clonal complexes from the data set are described below.

**The ST-11 complex Opa repertoire**

In the hyperinvasive ST-11 complex, the Opa repertoire was composed of allele 83 in the *opaA* locus, allele 11 in the *opaB* locus, allele 132 in the *opaD* locus and an insertionally inactivated *opaJ* locus. This repertoire was present in 27 of the 32 isolates (84.38) belonging to the ST-11 complex in the carried population sample. In the remaining 5 isolates, allele 354 was present in the *opaA* locus of 1 isolate, the sequence at the *opaB* locus of 2 isolates was insertionally inactivated and allele 18, which encoded identical variable regions to allele 11 was present in another. The sequence at the *opaD* locus of 1 isolate could not be determined. This repertoire was also present in 16 out of 20 isolates (80%) belonging to the ST-11 complex in the disease collection. This repertoire was similar to that observed in ST-11 meningococci from a geographically and temporally diverse collection of meningococci, with similar *opaB* (allele 11 in the carried population encoded identical HV regions to allele 34 in the global collection) and identical *opaD* loci [4].

**The ST-32 complex Opa repertoire**

The repertoires of the hyperinvasive ST-32 complex isolates in the carried, disease and global collections were almost identical, with *opaA96*, *opaB185*, *opaD147* and *opaJ218* present in the majority of isolates. The allele sequence of the *opaA* locus of 1 carried isolate but encoded the same variable regions as allele 96. Different alleles were present at the *opaB* loci of two isolates from the disease collection whereas the *opaD* and *opaJ* loci in the Czech isolates were identical to all but 1 of the ST-32 complex meningococci in the global collection.

**The ST-44 complex Opa repertoire**

In the hyperinvasive ST-41/44 complex, isolates belonging to individual sequence types had identical repertoires apart from those belonging to ST-44 itself, which exhibited higher alleleic diversity than other clonal complexes. There was a predominance of the HV1-19 family/HV2-11 family combination encoded by alleles at the *opaA* locus, alleles encoding the HV1-18 and HV2-15 families at the *opaB* locus and the HV1-1 family/ HV2-8 family combination at the *opaD* locus. In 22 out of 31 isolates belonging to this complex however, allele 213 was present at the *opaJ* locus.

**The ST-92 complex Opa repertoire**

This complex is not currently classified as hyperinvasive. In the *opaA* locus of the ST-92 complex 15 of 21 isolates had allele 94 (two isolates had allele 102, 1 had allele 13, two had different alleles encoding the same variable regions as one another, and one had a novel allele). At the *opaB* locus 14 of 21 isolates had allele 13 (3 had allele 17, 1 had an allele encoding identical HV regions, 2 had novel alleles and 1 had an allele generated from a potential recombination event involving allele 13 and allele 94). At the *opaD* locus, 16 of 21 isolates had allele 139 (1 isolate had an allele encoding identical variable regions to allele 139, 1 had a duplication of the allele at its *opaA* locus, 1 had an allele encoding identical HV regions and 2 had probable recombinationally generated alleles involving the allele at the *opaA* locus or the *opaB* locus and a novel allele). At the *opaJ* locus, 11 of 21 had allele 94 (1 isolate had the HV2-8B-2 variant rather than the 8B-1 variant found in allele 94, 1 allele encoded identical variable regions to allele 94, 2 had allele 224 which encoded different HV1-3 and HV2-8B variants to allele 94, 1 was the product of a potential recombination event between allele 94 and allele 159 at it’s *opaA* locus, four isolates had allele 13 the remaining isolate had a novel allele). This combination of *opaA94*, *opaB13*, *opaD139* and *opaJ94* accounted for the repertoires of just 5 of the 21 isolates however.

**The ST-106 complex Opa repertoire**

This complex is not currently classified as hyperinvasive. In the *opaA* locus of the ST-106 complex, a total of 12 of 19 isolates had allele 138 (1 had a duplication of allele 157, 1 was insertionally inactivated, two had duplications of the allele at their *opaB* locus and one had a novel allele); 14 of 19 isolates had allele 10 at the *opaB* locus (the other four had novel alleles); 15 of 19 isolates had allele 138 at the *opaD* locus (1 had a duplication of allele 157, 1 had 337 which encoded identical variable regions as allele 138 and two isolates had duplicated of the allele at their *opaA* locus) and but 1 isolate had allele 157 at the *opaJ* locus. This combination of *opaA138*, *opaB10*, *opaD138* and *opaJ157* accounted for the repertoires of just 10 of 19 isolates however.

1. *Simulations validating f* metric*

If the associations between loci were generated by means of a purely neutral, relatively random process, then one would expect an even distribution of allelic associations. Bacteria reproduce clonally, however, and the emergence and spread of successful clones means that certain combinations of alleles will dominate at any point in time. A purely random distribution of alleles would not adequately depict the distribution of alleles under neutral selection in a bacterial population, therefore. A stochastic individual-based model of strain evolution was used to generate hypothetical distributions of allelic associations and test the *f** statistic. Strains were defined by three loci; two antigenic and one housekeeping, each with five alleles (thus, strain space can be envisaged as a 5x5x5 matrix of allelic associations). The two antigenic loci determined host immunity, with hosts gaining a degree of protection against strains sharing alleles with a previously ‘seen’ strain dependent on γ, the level of cross-immunity. The housekeeping gene had no effect on the transmission of the strain, however it was subject to the same rates of mutation and recombination as the antigenic genes. All strains had exactly the same R_0_ value. Selection against overlapping antigenic associations should create a non-overlapping matrix of allelic associations between loci, however the associations between the antigenic loci and the housekeeping locus, as well as the associations between antigens when cross-immunity is low, should reflect the random pattern of clonal reproduction, strain transmission and recombination.

Simulations were run with different levels of cross-immunity until they had reached equilibrium, and the frequency of strains in the 5x5x5 matrix were then analysed. Other parameters were also varied, but the *f** statistic described above was insensitive to them, as expected. Figure 2 shows the distribution of *f** scores for simulations for antigen:antigen associations and for antigen:housekeeping associations for different levels of cross-immunity.


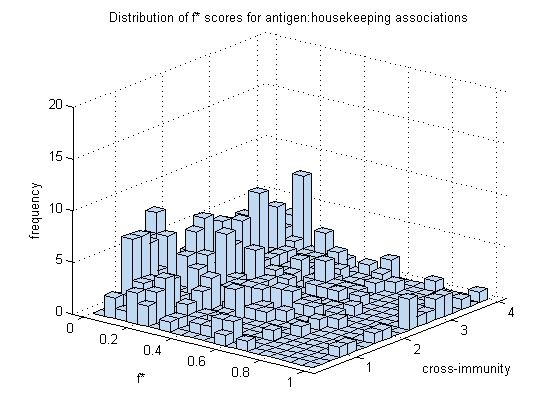

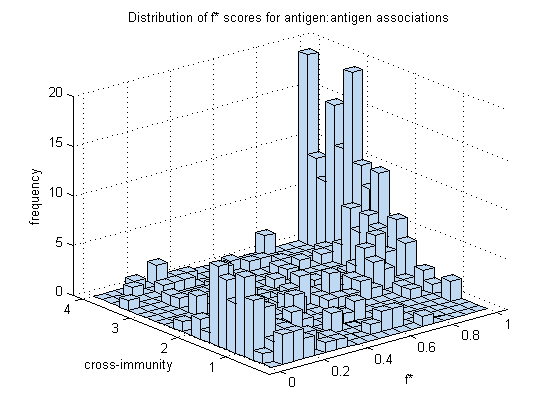


*Figure 2: Distributions of f* scores for different types of association (antigen and housekeeping above, antigen and antigen below) and different levels of cross-immunity for all simulations.*

For the antigen versus housekeeping gene comparison, the distribution of *f** scores is primarily low, since there is no immune selection on the housekeeping gene, and therefore no dependence on the level of cross-immunity. For antigenic loci however, as cross-immunity increases, there is increasing selection against strains which share alleles, and a non-overlapping structure emerges. This can be observed in the increasing *f** score for higher levels of cross-immunity, in the right-hand histogram.
