# Supplementary material for: The Effect of Immune Selection on the Structure of the Meningococcal Opa Protein Repertoire
Source: PLoS Pathog. 2008 Mar 14;4(3):e1000020. doi: 10.1371/journal.ppat.1000020 (PMC2265424; doi:10.1371/journal.ppat.1000020)
Supplement: Supplementary file 4 [file ppat.1000020.s003.doc]

HV1

HV2	1-6	8-B1	1-5	4-1	18-1	9-2	11-2	14-1	3-2	17-1	7-2	1-1	4-B1	15-2	5-2	6-1	1-4	12-3	10-3	17-3	17-6	2-1	4-3	8-C1	13-2	7-3	16-2	9-A1	16-1	11-1	8-A5	8-A4	19-1	21-1	3-4	1-2	9-3	1-7	8-A1	10-4	11-3	14-2	10-9	11-5	12-1	13-1	3-1	4-A5	4-B2	5-7	10-2	1-3	14-A	17-5	3-5	4-A1	4-A2	4-A3	4-B4	4-B6	5-1	5-4	5-5	5-9	8-A2	8-A3	8-B3	1-11	11-4	14-3	14-4	14-5	15-1	15-3	15-4	16-3	16-4	17-2	17-4	19-2	25-1	3-3	4-2	4-A4	4-A6	5-3	5-8	6-A1	8-1	8-2	8-B2	9-1	9-A2	Total
11-2	65	1																														3																																														1														70
3-5		30							1					1											7				2																																																									1		42
2-2			39																																																																1																									40
19-7				14									14																											2						3								1												1																						35
5-2					33																		1																																																																					34
6-1						28																	6																																																																					34
19-10							24						2																										4																											1	1		1																					33
18-3							1	31																																																																																					32
10-5									28																																					1							2																																						31
17-1										31																																																																																			31
4-2											27				1		3																																																																											31
3-6												27																																																																																	27
12-1													12																																		3										2	2														1		1					1	1								23
18-1							1						21																																																																															22
7-1															10																																														2	2	2																	1			1							18
1B-4																16																																																																													16
11-1																	11																																		2								2																																15
9-1																		11									4																																																																	15
10-1																			10																				3																																																					13
17-2																				8	5																																																																								13
19-1														1							7	5																																																																						13
16-2	7										2																				1													1																																														11
5-1																								10																																																								1												11
15-1																		1						5							1												3																																															10
4-1																										10																																																																			10
13-1																											5		2																								2																																							9
13-2																													8																																														1																	9
18-4							2																						7																																																															9
1-3																															8																																																														8
1B-3																																8																																																													8
12-2																																		7																																																											7
19-4				4																																					1															1																								1										7
9-3																																			6																																																										6
16-1																																				5																																																									5
19-3								1													4																																																																							5
19-9								5																																																																																					5
1-1																																					4																																																								4
15-2																																						3								1																																														4
1A-1					1																																	3																																																						4
1B-1		1		1																																		2																																																					4
5-4																								4																																																																					4
10-6																																											3																																																		3
14-1																																																								1	2																																				3
18-2																																																																									1	1		1																3
1A-3																																							2																																																		1			3
1A-4																																													3																																																3
1B-2																																																		3																																											3
10-4																																																			2																																										2
18-5																																																					2																																								2
19-8								2																																																																																					2
19-A1																														2																																																															2
19-A3																																												2																																																	2
1B-5																2																																																																													2
1B-6																																																																	2																												2
3-2																																																																		2																											2
3-4																																																																			2																										2
10-3																																																																																							1						1
11-3																	1																																																																												1
1-2																																					1																																																								1
12A-1												1																																																																																	1
14-2																																																								1																																					1
15-4																																																																																1													1
18-6								1																																																																																					1
18-8																																									1																																																				1
19-2																																										1																																																			1
19-6																																												1																																																	1
1A-2																																																																																									1				1
1-B8																																																																																													1	1
2-1			1																																																																																										1
3-1		1																																																																																											1
3-10		1																																																																																											1
4-3																																																																																												1		1
4-4												1																																																																																	1
5-3																																																																																								1					1
8-1																																					1																																																								1
9-2																		1																																																																											1
Total	72	34	40	18	35	28	28	40	28	32	27	31	12	38	10	20	12	16	10	8	5	11	5	21	5	10	5	11	10	9	8	8	7	7	6	5	6	3	5	5	5	4	3	3	3	3	3	3	3	3	2	2	2	2	2	2	2	2	2	2	2	2	2	2	2	2	2	1	1	1	1	1	1	1	1	1	1	1	1	1	1	1	1	1	1	1	1	1	1	1	1	1	1	1	784
